# Grb2 and GRAP connect the B cell antigen receptor to Erk MAP kinase activation in human B cells

**DOI:** 10.1038/s41598-018-22544-x

**Published:** 2018-03-09

**Authors:** Kanika Vanshylla, Caren Bartsch, Christoffer Hitzing, Laura Krümpelmann, Jürgen Wienands, Niklas Engels

**Affiliations:** University Medical Center Goettingen, Institute of Cellular & Molecular Immunology, Humboldtallee 34, 37073 Goettingen, Germany

## Abstract

The B cell antigen receptor (BCR) employs enzymatically inactive adaptor proteins to facilitate activation of intracellular signaling pathways. In animal model systems, adaptor proteins of the growth factor receptor-bound 2 (Grb2) family have been shown to serve critical functions in lymphocytes. However, the roles of Grb2 and the Grb2-related adaptor protein (GRAP) in human B lymphocytes remain unclear. Using TALEN-mediated gene targeting, we show that in human B cells Grb2 and GRAP amplify signaling by the immunoglobulin tail tyrosine (ITT) motif of mIgE-containing BCRs and furthermore connect immunoreceptor tyrosine-based activation motif (ITAM) signaling to activation of the Ras-controlled Erk MAP kinase pathway. In contrast to mouse B cells, BCR-induced activation of Erk in human B cells is largely independent of phospholipase C-ɣ activity and diacylglycerol-responsive members of Ras guanine nucleotide releasing proteins. Together, our results demonstrate that Grb2 family adaptors are critical regulators of ITAM and ITT signaling in naïve and IgE-switched human B cells.

## Introduction

Stimulation of the B cell antigen receptor (BCR) activates several intracellular signaling pathways that are executed by a coordinated interplay of several classes of enzymes and catalytically inert adaptor proteins^1^. Together with additional extracellular co-stimuli the amplitude and kinetics of BCR-induced signaling determine the differentiation fate of an individual B lymphocyte^2^. The canonical signal-activating elements of the BCR are two copies of the immunoreceptor tyrosine-based activation motif (ITAM), which are present in the cytoplasmic domains of the BCR signaling subunits Igα (CD79A) and Igβ (CD79B)^3^. Phosphorylation of ITAMs creates binding sites for Src homology 2 (SH2) domain-containing protein tyrosine kinases (PTKs) of the Src and Syk/ZAP70 families^4,5^. In B cells, ITAM-bound Syk phosphorylates the central adaptor protein SH2 domain-containing adaptor protein of 65 kDa (SLP65, alternatively called BLNK), which is also recruited to the BCR by binding with its SH2 domain to a phosphorylated non-ITAM tyrosine (Y204) in Igα^6–9^. In its phosphorylated state SLP65 recruits the PTK Bruton´s tyrosine kinase (Btk) and phospholipase C-ɣ2 (PLC-ɣ2) via their SH2 domains to form the so-called Ca^2+^ initiation complex, in which Btk phosphorylates and activates PLC-ɣ2^10^. Activated PLC-ɣ2 hydrolyzes the plasma membrane phospho-lipid phosphatidyl-inositol-4,5-bisphosphate, resulting in the generation of two critical second messengers: membrane-resident diacylglycerol (DAG) and soluble inositol-1,4,5-trisphosphate (IP3). IP3 triggers opening of a ligand-gated Ca^2+^ channel in the membrane of the endoplasmic reticulum called IP3 receptor, which results in a transient rise in cytosolic Ca^2+^ concentrations. This first wave of Ca^2+^ entry into the cytosol is followed by a second wave that is brought about by opening of Ca^2+^ channels in the plasma membrane^11^. This canonical pathway of BCR-induced Ca^2+^ mobilization is employed by all BCR isotypes, since every membrane-bound immunoglobulin (mIg) isoform associates with the invariant Igα/β heterodimer to form a fully assembled, functional BCR complex. However, BCR-induced Ca^2+^ mobilization can be strongly amplified by a signaling motif that is found in the cytoplasmic tails of mIgG and mIgE^12^. In mIgG, this immunoglobulin tail tyrosine (ITT) motif was shown to recruit the adaptor protein growth factor receptor-bound 2 (Grb2), which in turn brings Btk directly to the activated mIgG-BCR to facilitate activation of PLC-ɣ2 in memory B cells and thus promotes production of IgG antibodies *in vivo*^13,14^. Whether mIgE-containing BCRs employ the same mechanism or have evolved additional ways to amplify activation of PLC-ɣ2 remains unclear.

In the context of BCR signaling, Grb2 was initially reported to have an inhibitory impact since it subdued Ca^2+^ mobilization in the chicken B cell line DT40 as well as in primary mouse B cells on stimulation of mIgM-containing BCRs^15–17^. This signal-attenuating function of Grb2 required its SH2 domain-mediated association with the negative-regulatory adaptor protein docking protein 3 (Dok3) or the inhibitory cell surface receptor CD22^18,19^. Hence, Grb2 can fulfil either amplifying or inhibitory effects on BCR-induced second messenger production, depending most likely on the signaling microenvironment it is recruited to (ITT motifs or inhibitory components). Besides Grb2, B cells express another member of this family of adaptor protein termed Grb2-related adaptor protein (GRAP), which in contrast to the ubiquitously expressed Grb2 is selectively found in hematopoietic cells. GRAP shares the SH3-SH2-SH3 domain architecture with Grb2 and like Grb2 constitutively associates with the guanine nucleotide exchange factor (GEF) son of sevenless (Sos)^20,21^. However, whether GRAP is involved in BCR signaling remains unclear.

A key signaling pathway that is used not only by the BCR but by many different receptors to control cellular proliferation, survival and differentiation is the extracellular signal regulated kinase (Erk) mitogen activated protein (MAP) kinase pathway. In B cells, the Erk-MAP kinase pathway has been shown to be required for progression of early B cell development and the establishment and maintenance of memory B cells^22–27^. The Erk MAP kinase pathway is controlled by the activity of the ubiquitous small G protein Ras^28^. Ras itself is under tight control and mutations causing its constitutive activation are among the most frequent mutations in many human cancer entities, demonstrating the impact of this pathway on cell proliferation^29,30^. Ras activity is balanced by two classes of regulatory proteins: GEFs that activate Ras by catalyzing the replacement of Ras-bound GDP for GTP, and GTPase-activating proteins (GAPs), which serve as essential co-factors in the GTP hydrolysis reaction that causes Ras to switch back to its GDP-bound inactive state^31^. The biochemical events leading to activation of Ras following BCR stimulation were studied mostly in mouse models or in a B cell line derived from chicken. In both model systems the BCR activates Ras via a small family of GEFs referred to as Ras guanine-nucleotide releasing proteins (RasGRPs)^32^. In particular the DAG-binding isoforms RasGRP1 and RasGRP3 were shown to control activation of Ras and the Erk MAP kinase pathway in these animal B cells^33–35^. However, there is an alternative and more ubiquitous mechanism of Ras activation that involves the GEF Sos and its constitutive binding partner Grb2^36^. In this scenario, which has been well established for receptor tyrosine kinases (RTKs) such as many growth factor receptors, Grb2 docks with its SH2 domain to conserved tyrosine phosphorylation motifs within the cytoplasmic domains of activated RTKs and brings Sos ‘piggyback’ to the plasma membrane, where it meets and activates membrane-anchored Ras proteins^37^. Which of the two routes leading to activation of Ras and Erk operates in human B cells remains unknown.

Here, we investigated the role of Grb2 and GRAP in human B cells and found that both adaptor proteins can amplify Ca^2+^ mobilization and activation of the Erk MAP kinase pathway by the ITT motif of mIgE-BCRs. Furthermore, in human B cells Grb2 and GRAP are required for activation of Erk by the canonical ITAM pathway as well. In contrast to mouse B cells, mature human B cells express only very small amounts of DAG-responsive RasGRP isoforms and are less dependent on PLC-ɣ activity to couple BCR engagement to Erk activation. Instead BCRs in human B cells seem to employ the Grb2(GRAP)/Sos axis in a manner similar to that of receptor tyrosine kinases.

## Results

### Grb2 and GRAP amplify mIgE-BCR signaling

**Figure 1:**
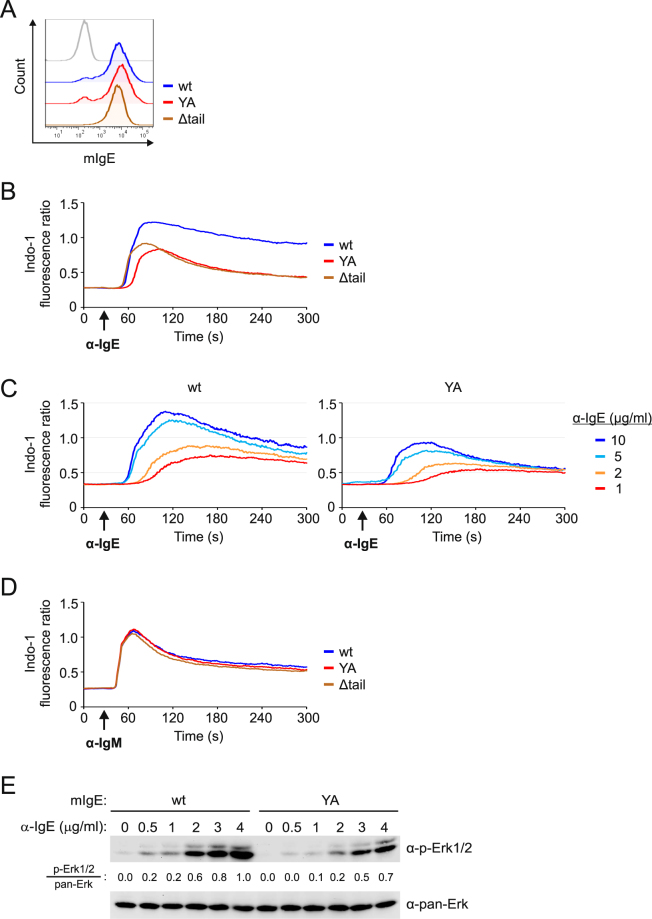
The ITT motif in membrane-bound IgE amplifies BCR-induced Ca^2+^ mobilization and activation of Erk. DG75EB cells were retrovirally transduced to express either wild type (wt), an ITT tyrosine to alanine mutant (YA) or a truncated version of mIgE lacking the C-terminal 24 amino acids of the cytoplasmic domain (Δtail). Surface expression of the mIgE variants in sorted cells is shown in (**A**). (**B**) BCR-induced mobilization of Ca^2+^ ions was analyzed on stimulation of the cells with 10 µg/ml anti-IgE antibodies. (**C**) BCR-induced Ca^2+^ mobilization of cells expressing either wild type or YA-mutant mIgE-BCRs using the indicated concentrations of anti-IgE antibodies. (**D**) The same cells were stimulated with 20 µg/ml anti-IgM F(ab’)_2_ fragments as control. (**E**) Activation of Erk kinases following stimulation of wild type or ITT-mutant mIgE-BCRs was analyzed by immunoblotting of cleared cellular lysates using antibodies to phospho-Erk (α-p-Erk1/2) and non-phosphorylated Erk (α-pan-Erk). Band intensities were quantified and the ratio of signal intensities for phospho-Erk divided by the signal intensities for total Erk is given. Maximal Erk activation was set to 1.0 and all other signal intensities were calculated accordingly. Data are representative of three independent experiments.

The adaptor protein Grb2 has a positive role in signaling of mIgG-BCRs^13^. However, whether the ITT motifs of mIgG and mIgE are functionally identical or employ distinct sets of effector proteins remains incompletely understood. Previous reports suggested that the ITT-like motif in mIgE may enhance mIgE-BCR internalization by recruiting the cytoplasmic adaptor protein HAX1 or may be involved in preventing inhibitory signals by CD22^38,39^. However, both reports postulated a phosphorylation-independent function of the mIgE ITT motif. We analyzed the impact of phosphorylation of the ITT motif on connecting the mIgE-BCR to intracellular signaling pathways. To this end we generated mutant variants of the human εm immunoglobulin (Ig) heavy chain in which the central ITT tyrosine residue was replaced by an alanine (YA) or the cytoplasmic tail was shortened to four amino acids (Δtail). Wild type and mutant εm Ig heavy chains were retrovirally expressed in the human B cell line DG75 and the transfectants were sorted for equal expression of surface mIgE (Fig. 1A). Stimulation of the different mIgE-BCR variants with antibodies specific for human (m)IgE (Supplementary Fig. 1) showed that the ITT motif strongly enhanced intracellular Ca^2+^ mobilization. Deletion of the entire cytoplasmic domain had no additional adverse effect on Ca^2+^ signaling, showing that the ITT is the only motif within the mIgE tail that amplifies PLCɣ activity and subsequent Ca^2+^ mobilization (Fig. 1B). By titrating the concentration of the stimulating anti-IgE antibody we could show that the ITT amplifies Ca^2+^ signaling even under suboptimal stimulation conditions (Fig. 1C) and thus enhances the sensitivity of mIgE-BCRs to antigen. To test the general signaling capacities of the different transfectants, we measured Ca^2+^ mobilization of the transfected cells on stimulation of their endogenous mIgM-BCRs, which resulted in equal Ca^2+^ mobilization kinetics in all cells and thus demonstrated that the transfectants were not corrupted in any way (Fig. 1D). We also tested the effect of ITT phosphorylation on activation of the Erk MAP kinase pathway using various concentrations of stimulating anti-IgE antibody (Fig. 1E and Supplementary Fig. 2). Again the results showed that the ITT motif amplifies the sensitivity of mIgE-BCRs since it strongly enhanced activation of Erk, especially when using suboptimal doses of stimulating reagent. Similar results were obtained when the experiments were made in the human B cell line Ramos (Supplementary Fig. 3).

**Figure 2:**
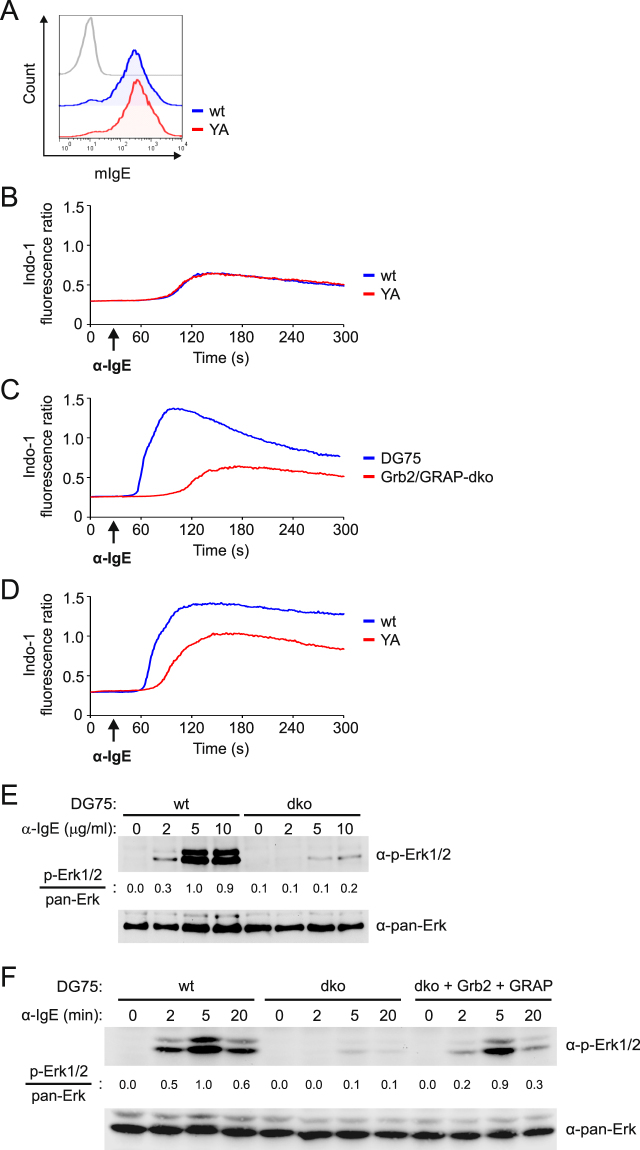
The ITT of mIgE-BCRs employs Grb2 and GRAP for signal amplification. DG75 cells deficient for Grb2 and GRAP were retrovirally transduced to express either wild type (wt) or ITT-mutant (YA) mIgE. Surface expression of mIgE variants is shown in (**A**), their Ca^2+^ mobilization profiles on stimulation with 10 µg/ml anti-IgE antibodies are shown in (**B**). (**C**) Ca^2+^ mobilization kinetics of wild type mIgE-BCRs in parental DG75 cells (blue curve) and Grb2/GRAP double-deficient cells (Grb2/GRAP-dko, red curve). (**D**) Grb2/GRAP double-deficient cells expressing wild type or ITT-mutant mIgE (from (**A**) and (**B**)) were additionally transduced to express both Grb2 along with EFGP and GRAP together with tagRFP. Ca^2+^ mobilization kinetics of the cells on stimulation of mIgE-BCRs was analyzed as before. (**E**) DG75 B cells (wt) and Grb2/GRAP double-deficient cells (dko) expressing wild type mIgE-BCRs were stimulated with the indicated concentrations of anti-IgE antibodies for five minutes. Phosphorylation of Erk proteins was analyzed by immunoblotting and relative band intensities were determined as before. (**F**) IgE-BCR-induced time course of Erk phosphorylation in DG75 (wt), Grb2/GRAP double-deficient cells (dko) and double-deficient cells reconstituted to express Grb2 and GRAP (dko + Grb2 + GRAP). Data are representative of three independent experiments.

The universal adaptor protein Grb2 binds to the ITT motif of mIgG-BCRs. This interaction requires the SH2 domain of Grb2, which interacts with the (phospho-) tyrosine-X-asparagine (YxN) ITT core sequence motif of mIgG isotypes. In previous studies we found that Grb2 is essential for ITT-mediated signal amplification of mIgG-BCRs^13^. However, when we expressed mIgE or chimeric mIgG molecules containing the intracellular domain of reptilian mIgY (mIgG/Y) in Grb2-deficient B cells, the ITT motifs of these mIg isotypes were still capable of amplifying BCR-induced Ca^2+^ mobilization, albeit to a reduced extent (Supplementary Fig. 4). These findings indicated that the ITT motifs of mIgE and mIgY do not exclusively signal via Grb2 but can employ additional effector proteins. The ITTs of mIgE and mIgY contain the same core motif, yet the surrounding amino acid sequences are different from that of the mIgG-ITT motif (Supplementary Fig. 5A). To identify interaction partners of the mIgE-ITT that might exert its signal-amplifying effect, we used a phosphorylated peptide encompassing the ITT motif of human εm for affinity purification of proteins from lysates of human B cells. To get quantitative information on the ITT interactions, we used the SILAC method in which the cells are metabolically labeled with stable isotopes of carbon and nitrogen (provided within the amino acids arginine and lysine in the medium) and used a non-phosphorylated peptide and non-labeled cells as control (Supplementary Fig. 5B and C). Among the most efficiently enriched proteins were the universal cytosolic adaptor proteins Grb2 and GRAP, both of which are known to bind with their SH2 domains to phosphorylated tyrosine motifs with the consensus sequence phospho-YxN, which is present in the mIgE-ITT (Supplementary Fig. 5D and E). Additional biochemical interaction studies verified the direct interaction of the two adaptor proteins with the mIgE-ITT (Supplementary Fig. 6). Replacement of the asparagine residue in the ITT motif with alanine (NA) does not interfere with phosphorylation of the tyrosine residue, but specifically prevents binding of Grb2 and GRAP. Clearly, also this amino acid substitution in mIgE renders the ITT non-functional (Supplementary Fig. 7). Hence, we considered Grb2 and GRAP the most obvious candidates in connecting the ITT to downstream signaling pathways. To test this hypothesis, we generated a Grb2/GRAP double-deficient variant of DG75 B cells (Supplementary Fig. 8). Please note that our TALEN-based approach to genetically inactivate GRAP in the Grb2-deficient background did not yield clones in which the *GRAP* open reading frame was disrupted. However, we were able to identify a clone in which deletions of three and six codons per allele caused a virtually complete loss of GRAP protein expression (Supplementary Fig. 8C). These cells, which are referred to as Grb2/GRAP double-deficient cells hereafter, were retrovirally transfected to express wild type or ITT-YA-mutant mIgE as before (Fig. 2A) and analyzed for mIgE-BCR-induced Ca^2+^ mobilization. Indeed, the absence of both Grb2 and GRAP not only rendered the mIgE-ITT completely non-functional (Fig. 2B), but also resulted in a drastic reduction of mIgE-BCR-induced Ca^2+^ mobilization as compared to wild type DG75 cells (Fig. 2C). Reconstitution of the Grb2/GRAP double-deficient cells with either Grb2 or GRAP or both, either partially or completely restored the signal-amplifying effect of the ITT (Fig. 2D and Supplementary Fig. 9). We also tested the capacity of the mIgE-BCR to activate the Erk MAP kinase pathway in Grb2/GRAP double-deficient cells and found that even under optimal stimulation conditions, the mIgE-BCR could not activate Erk in the absence of the two adaptor proteins (Fig. 2E and Supplementary Fig. 10A). Again, re-expression of Grb2 and GRAP restored the signaling defect (Fig. 2F and Supplementary Fig. 10B), showing that Grb2-family adaptor proteins are critically involved in Erk MAP kinase activation in DG75 B cells.

### PLCɣ-independent activation of the Erk MAP kinase pathway in human B cells

**Figure 3:**
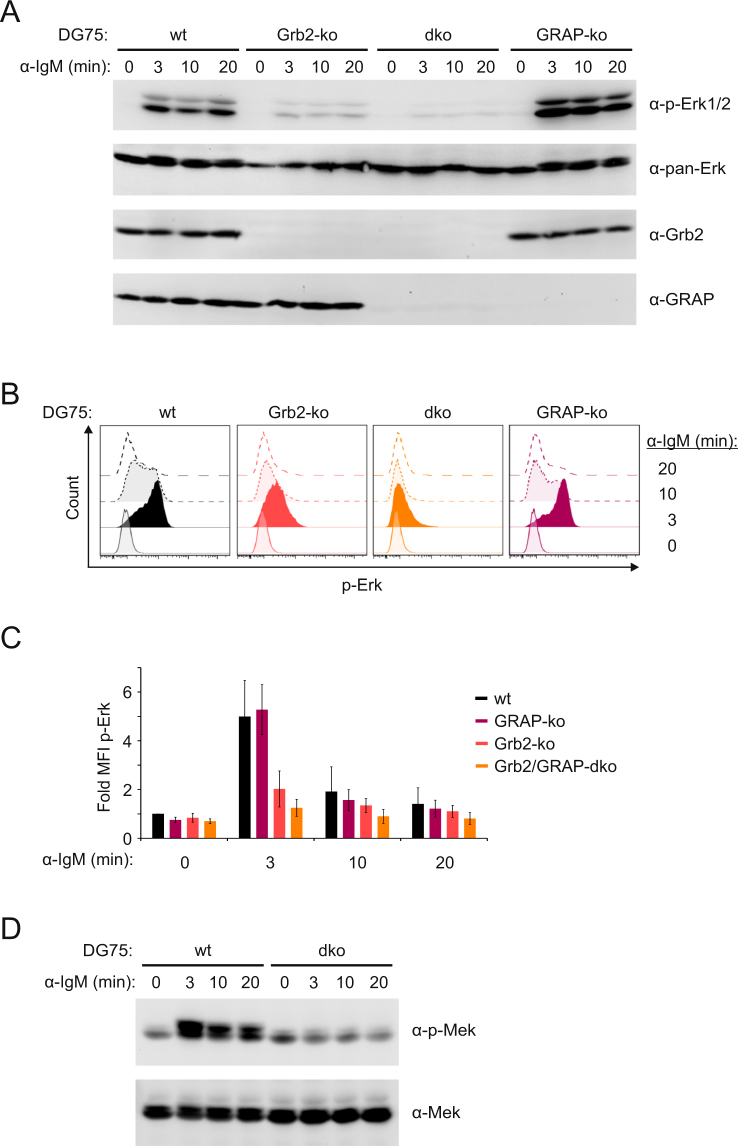
Grb2 and GRAP are essential for activation of Erk by the mIgM-BCR in DG75 B cells. (**A**) Activation of Erk in DG75 cells (wt), and variants lacking either Grb2 (Grb2-ko) or GRAP (GRAP-ko) or both (dko) following stimulation with 20 µg/ml anti-IgM F(ab’)_2_ fragments for the indicated times. Cleared cellular lysates were analyzed as before. In addition the expression of Grb2 and GRAP in the different DG75 sublines was tested with the indicated antibodies. (**B**) Quantitative analysis of Erk phosphorylation in the same cells using intracellular staining of phospho-Erk followed by flow cytometric analysis of mean fluorescence intensities (MFIs). The average changes in MFIs of three independent experiments are shown in (**C**). Basal signal intensities of unstimulated parental DG75 cells (wt) were defined as 1.0 and all other intensities were normalized accordingly. Error bars represent standard deviation of the mean of three independent experiments. (**D**) Parental DG75 cells (wt) and Grb2/GRAP double-deficient cells (dko) were stimulated for the indicated times with 20 µg/ml anti-IgM F(ab’)_2_ fragments. Cleared cellular lysates were analyzed for phosphorylated Mek (α-p-Mek) and total Mek (α-Mek) as loading control. Data are representative of three independent experiments.

**Figure 4:**
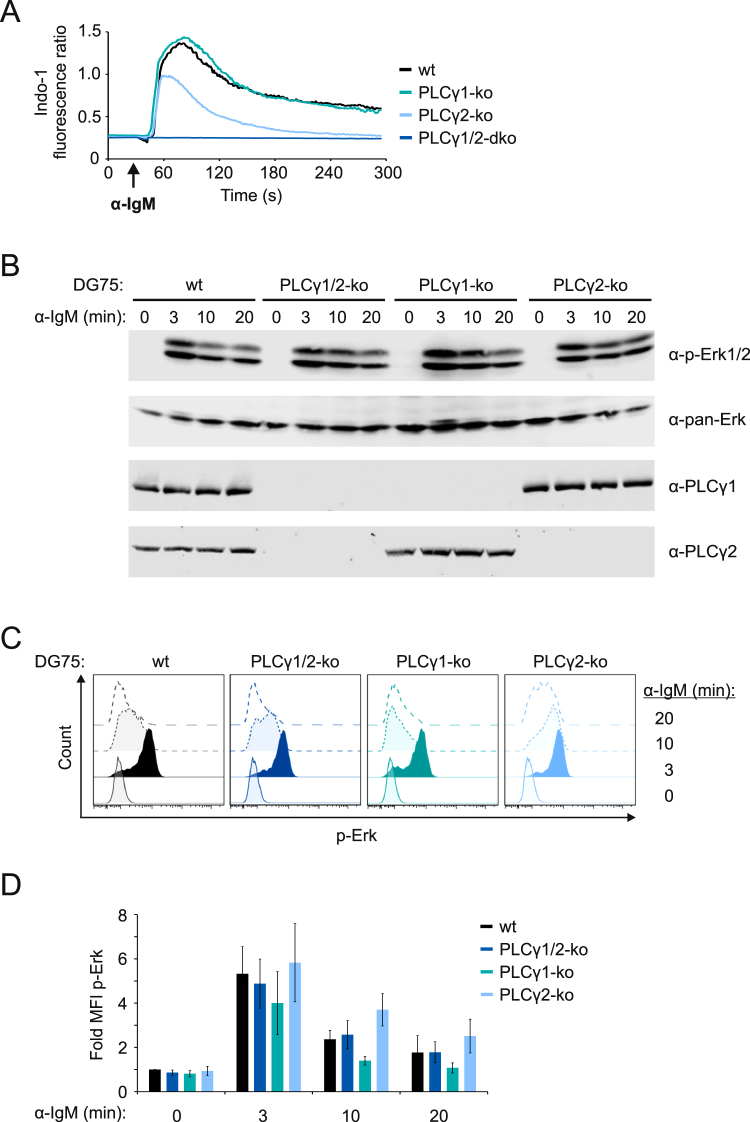
PLCɣ isoforms are not required for activation of Erk in DG75 B cells. (**A**) Ca^2+^ mobilization profiles of parental DG75 cells (wt, black curve) and sublines lacking either PLCɣ1 (turquoise curve) or PLCɣ2 (light blue curve) or both (dark blue curve) upon stimulation of the endogenous mIgM-BCR with 20 µg/ml anti-IgM F(ab’)_2_ fragments. (**B**) The kinetics of Erk activation in the same cells was analyzed by immunoblotting (**B**) and intracellular staining (**C**) as before. The average changes in MFIs of three independent experiments are shown in (**D**). Basal signal intensities of unstimulated parental DG75 cells (wt) were defined as 1.0 and all other intensities were normalized accordingly. Error bars represent standard deviation of the mean of three independent experiments.

In mouse and chicken B cells the Erk pathway is under control of phospholipase Cɣ2 (PLCɣ2) activity and requires the subsequent recruitment of RasGRP isoforms to the plasma membrane via binding to the PLCɣ2 product diacylglycerol (DAG)^33,34^. Hence, the strongly diminished Ca^2+^ mobilization kinetics in Grb2/GRAP double-deficient cells could account for the inability of the mIgE-BCR to activate the Erk pathway (see Fig. 2C). However, also the endogenous mIgM-BCR of DG75 B cells failed to activate the Erk pathway in in the absence of Grb2 and GRAP as measured by immunoblot analysis as well as intracellular staining of phosphorylated Erk (Fig. 3A–C and Supplementary Fig. 11A). Also the Erk-activating kinase Mek was not phosphorylated in the absence of Grb2 and GRAP (Fig. 3D and Supplementary Fig. 11B), despite robust Ca^2+^ mobilization in these cells on stimulation of the endogenous mIgM-BCR (Supplementary Fig. 8E). These observations raised the question as to whether or not PLCɣ is involved in BCR-induced activation of the Erk MAP kinase pathway in human B cells at all. To address this question we generated DG75 B cells that are deficient for both PLCɣ2 and PLCɣ1 by TALEN mutagenesis (Supplementary Figs 12 and 13). The PLCɣ1/2 double-deficient cells did not show any Ca^2+^ mobilization on BCR stimulation (Fig. 4A) but normal Erk phosphorylation kinetics (Fig. 4B–D and Supplementary Fig. 14). To verify this result in an independent genetic model system, we generated B cells deficient for the central BCR adaptor protein SLP65, using TALEN-mediated genome-editing as before (Supplementary Fig. 15). Like the PLCɣ1/2 double-deficient cells, the SLP65-deficient cells did not show BCR-induced Ca^2+^ mobilization but normal phosphorylation of Erk (Supplementary Fig. 1^6^). Together, these results demonstrate that activation of the Erk MAP kinase pathway can occur in human B cells via a mechanism that is independent of PLCɣ activity and thus independent of DAG production.

### PLCɣ-independent activation of the Erk MAP kinase pathway in primary human mIgM-expressing B cells

**Figure 5:**
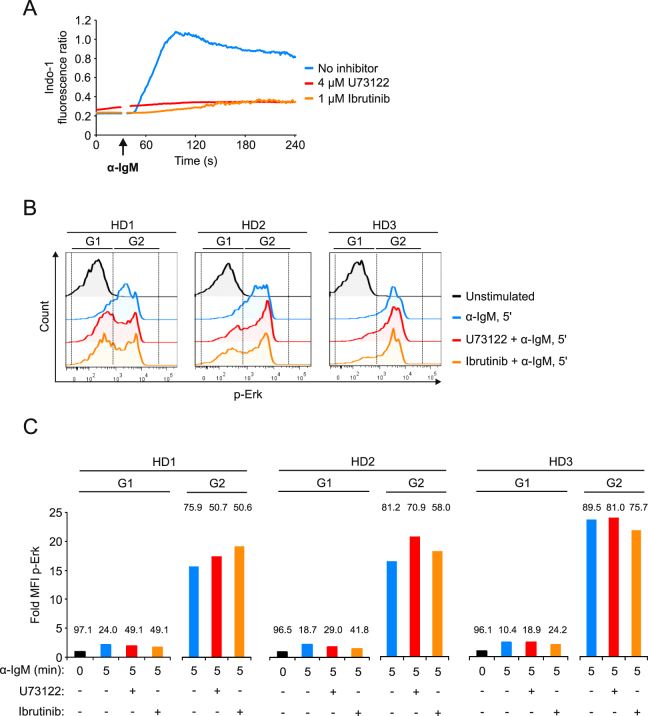
Phosphorylation of Erk in primary human B cells is largely independent of PLCɣ-activity. (**A**) The Ca^2+^ mobilization kinetics of primary human B cells isolated from peripheral blood of healthy donor 1 were analyzed following stimulation with 15 µg/ml anti-IgM F(ab’)_2_ fragments in the absence (blue curve) or the presence of either 4 µM U73122 (red curve) or 1 µM Ibrutinib (orange curve). (**B**) Phosphorylation of Erk in primary human peripheral blood B cells from three healthy donors (HD1–3) following stimulation of mIgM-BCRs for five minutes was analyzed by intracellular staining of phospho-Erk and flow cytometry as before. Cells were stimulated in the absence (blue curves) or presence of 4 µM U73122 (red curves) or 1 µM Ibrutinib (orange curves). The peak of unstimulated cells was used to define gate 1 (G1), the peak of BCR-stimulated cells of healthy donor 3 (HD3) was used to define gate 2 (G2). (**C**) Bar diagram of normalized mean fluorescence intensities shown in (**B**). Black bars represent unstimulated cells, blue bars represent cells that were stimulated without inhibitor, red and orange bars represent cells that were stimulated in the presence of either 4 µM U73122 or 1 µM Ibrutinib, respectively. Basal signal intensities of unstimulated cells from the respective donors were defined as 1.0 and all other intensities were normalized accordingly. Numbers above bars indicate the percentage of cells in the respective gate. Data shown are from one experiment that is representative for three independent experiments.

To test whether the findings we made in DG75 B cells regarding the dispensability of PLCɣ activity for connecting the Erk MAP kinase pathway to BCR activation are representative for primary human B cells, we used two pharmacological inhibitors to block PLC-ɣ activity and thus the generation of IP3 and DAG in B cells from peripheral blood of three healthy donors. U73122 is a direct inhibitor of PLC isoforms, whereas Ibrutinib is an inhibitor of the PLC-ɣ-activating kinase Btk. We titrated the minimum effective concentrations of the inhibitors in primary human B cells using Ca^2+^-mobilization as read out for PLCɣ activity and found that 4 µM U73122 and 1 µM Ibrutinib effectively blunt BCR-induced Ca^2+^ flux (Fig. 5A). Next, we analyzed BCR-induced Erk activation in primary human B cells in the absence or presence of the inhibitors using intracellular staining of phospho-Erk and flow cytometry as before (Fig. 5B). B cells of three healthy donors (HD) were stimulated with anti-IgM F(ab’)_2_ fragments and identified by staining with antibodies to CD19 and IgM (Supplementary Fig. 17). This approach revealed that there are two populations of mIgM-expressing B cells in human blood that differ in their sensitivity to the inhibitors. Whereas one population showed reduced Erk phosphorylation in the presence of U73122 or Ibrutinib (gate G1 in Fig. 5B and red and orange bars from G1 in Fig. 5C), another population, which contained approximately 50–75% of all mIgM-positive peripheral blood B cells (gate G2 in Fig. 5B and red and orange bars from G2 in Fig. 5C), was completely resistant to these inhibitors. Importantly, in each donor we observed a population containing roughly 10–25% of mIgM-expressing cells that showed a very poor phosphorylation of Erk even in the absence of the inhibitors (blue bars from G1 in Fig. 5C). Thus, of those cells that did respond to the stimulus with phosphorylation of Erk, the vast majority did not require PLCɣ activity. In conclusion the observations we made in DG75 B cells regarding the dispensability of PLCɣ activity for BCR-induced activation of the Erk MAP kinase pathway are representative for a large if not the predominant population of mIgM-expressing B cells found in human blood.

### The expression of DAG-responsive RasGRP isoforms is very low in primary human B cells

**Figure 6:**
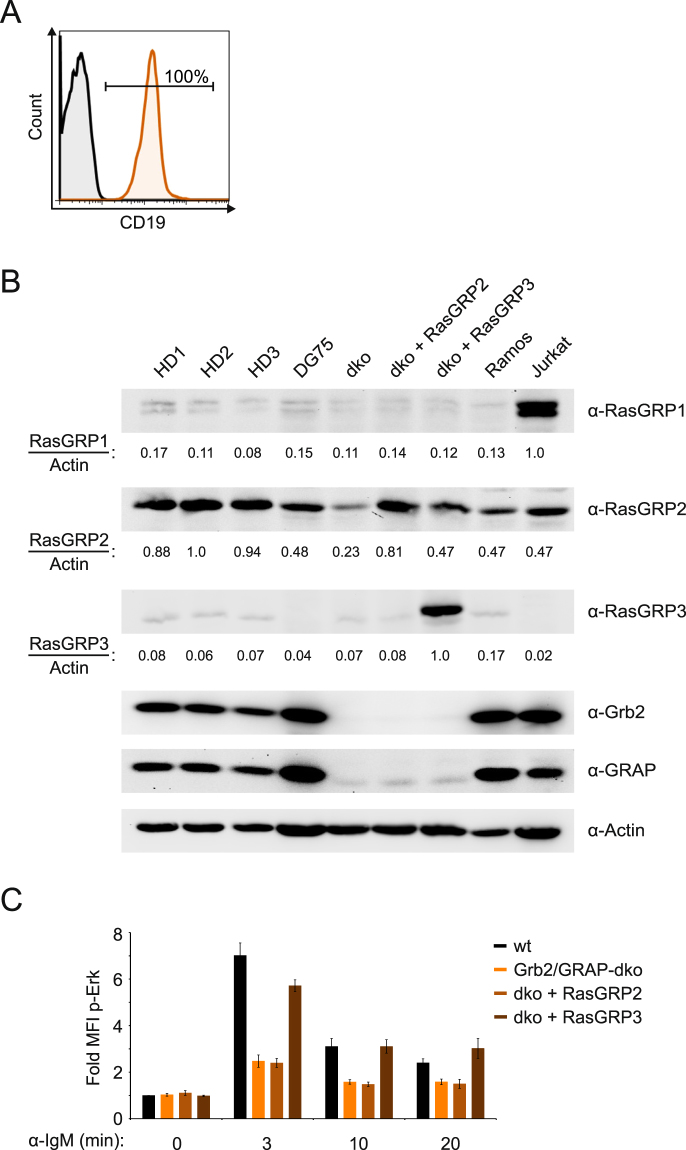
DAG-responsive RasGRP isoforms are poorly expressed in human B cells. (**A**) Primary human B cells isolated from peripheral blood of a healthy donor were stained with anti-CD19 antibodies to verify the purity of the used cells. (**B**) Cleared cellular lysates of primary human B cells, the Burkitt lymphoma lines Ramos and DG75 and the human T cell line Jurkat were prepared. In addition, Grb2/GRAP double-deficient DG75 cells (Grb2/GRAP-dko) were retrovirally transduced to express RasGRP2 or RasGRP3, respectively, along with IRES-driven EFGP and sorted for EGFP expression. Lysates of all cell types were loaded onto two gels and blotted simultaneously. One membrane was probed with antibodies to RasGRP isoforms 1 and 2, Grb2 and GRAP, the second membrane was probed with antibodies to RasGRP3. Both blots were probed with anti-β-Actin as loading control. The relative band intensities for RasGRP isoforms normalized to the respective Actin signals are shown below the blots. Data are representative of three independent experiments. (**C**) Quantification of Erk phosphorylation in the same cells was measured by phos-flow analysis as before. Basal signal intensities of unstimulated parental DG75 cells (wt) were defined as 1.0 and all other intensities were normalized accordingly. Error bars represent standard deviation of the mean of three independent experiments.

Murine B cells were shown to depend on PLCɣ activity and DAG-responsive RasGRP isoforms to couple BCR stimulation to activation of the Erk MAP kinase pathway^33^. Since in both DG75 as well as in the majority of primary human B cells PLCɣ activity is not essential for this process, we tested whether RasGRP family members are expressed in human B cells by immunoblot analysis using cellular lysates of freshly isolated peripheral blood B cells (Fig. 6A). As controls we included the human Burkitt lymphoma cell line Ramos and the T cell line Jurkat (which were previously shown to express RasGRP3 and RasGRP1, respectively). Both DG75 B cells as well as primary human B cells showed an almost identical expression pattern of RasGRP family members in that they hardly express any RasGRP1 and RasGRP3, yet contain considerable amounts of RasGRP2 (Fig. 6B and Supplementary Fig. 18). Also they express similar amounts of Grb2 and GRAP. Hence, we conclude that DG75 cells mirror the situation in primary human B cells. However, since DG75 B cells express a little less RasGRP2 than primary human B cells, we ectopically expressed additional RasGRP2 in Grb2/GRAP double-deficient DG75 cells to achieve an expression level of RasGRP2 similar to that found in primary cells (Fig. 6B, dko + RasGRP2). However, even ectopic expression of RasGRP2 could not restore BCR-induced activation of the Erk pathway in the absence of Grb2 and GRAP (Fig. 6C). To test, whether DG75 B cells can use RasGRP proteins to mediate activation of the Erk MAP kinase pathway at all, we expressed RasGRP3 in the Grb2/GRAP double-deficient cells and analyzed Erk activation as before (Fig. 6B,C, dko + RasGRP3). The results demonstrate that DG75 B cells are capable of using DAG-responsive RasGRP family members to connect PLCɣ activity to activation of the Erk MAP kinase pathway and thus once more prove that the DG75 B cell line is a valid model system to study the roles of Grb2 adaptors and RasGRP family proteins for activation of the Erk MAP kinase pathway in human B cells.

### Differential requirement of Grb2 interaction domains for Erk activation and mIgE-BCR signaling

**Figure 7:**
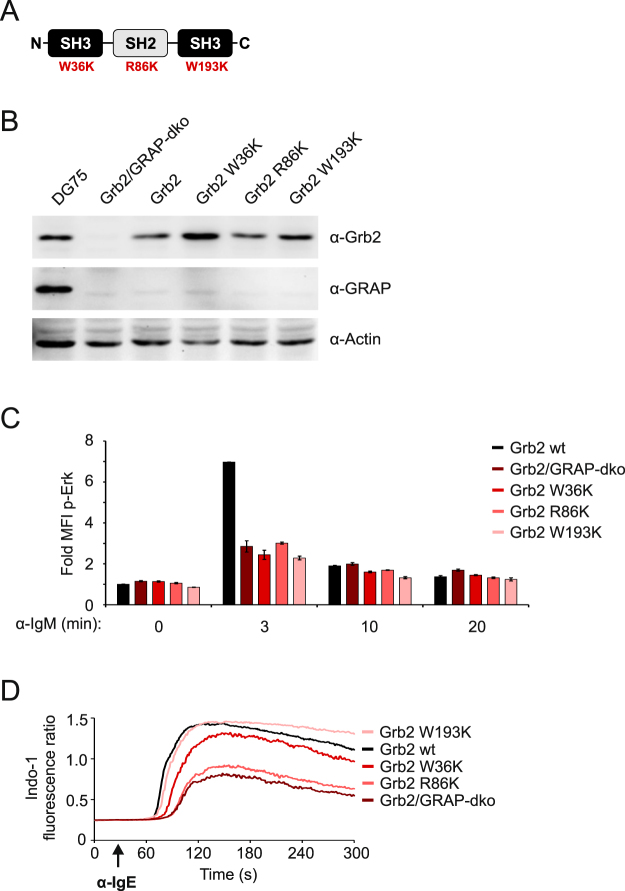
Grb2 domains cooperate in activation of Erk in human B cells. (**A**) Domain organization of Grb2. The amino acid substitutions that were introduced to inactivate the respective domains are given in red. (**B**) The expression of the various Grb2 variants in DG75 Grb2/GRAP-dko cells was analyzed by immunoblotting. (**C**) Quantification of Erk phosphorylation in the same cells was measured by phos-flow analysis as before. Basal signal intensities of unstimulated parental DG75 cells (wt) were defined as 1.0 and all other intensities were normalized accordingly. Error bars represent standard deviation of the mean of three independent experiments. (**D**) Ca^2+^ mobilization kinetics of the same cells stimulated via the mIgE-BCR.

Having established that a substantial population of human B cells does not require PLCɣ activity but most likely employs Grb2 family adaptor proteins to connect the activated BCR to the ubiquitous Erk MAP kinase pathway, we investigated the molecular requirements of that process in more detail. Specifically, we expressed mutant variants of Grb2, in which individual SH2 or SH3 interaction domains were inactivated, in Grb2/GRAP double-deficient DG75 cells (Fig. 7A,B and Supplementary Fig. 19) and tested their ability to mediate BCR-induced Erk activation. These experiments revealed that all three domains of Grb2 are equally required to induce proper activation of the Erk pathway, since inactivation of either SH domain abolished phosphorylation of Erk kinases on stimulation of the mIgM-BCR and the mIgE-BCR alike (Fig. 7C and Supplementary Fig. 20). Interestingly, the only mutation in Grb2 that abrogated ITT-mediated Ca^2+^ amplification downstream of mIgE-BCRs was the R86K substitution, which inactivates the SH2 domain (Fig. 7D). Hence Grb2 uses different ways to connect the BCR to activation of the Erk MAP kinase pathway and amplification of Ca^2+^ mobilization, respectively, demonstrating once more that these two pathways are not connected in human B cells.

### Grb2 is directly recruited to the phosphorylated BCR

**Figure 8:**
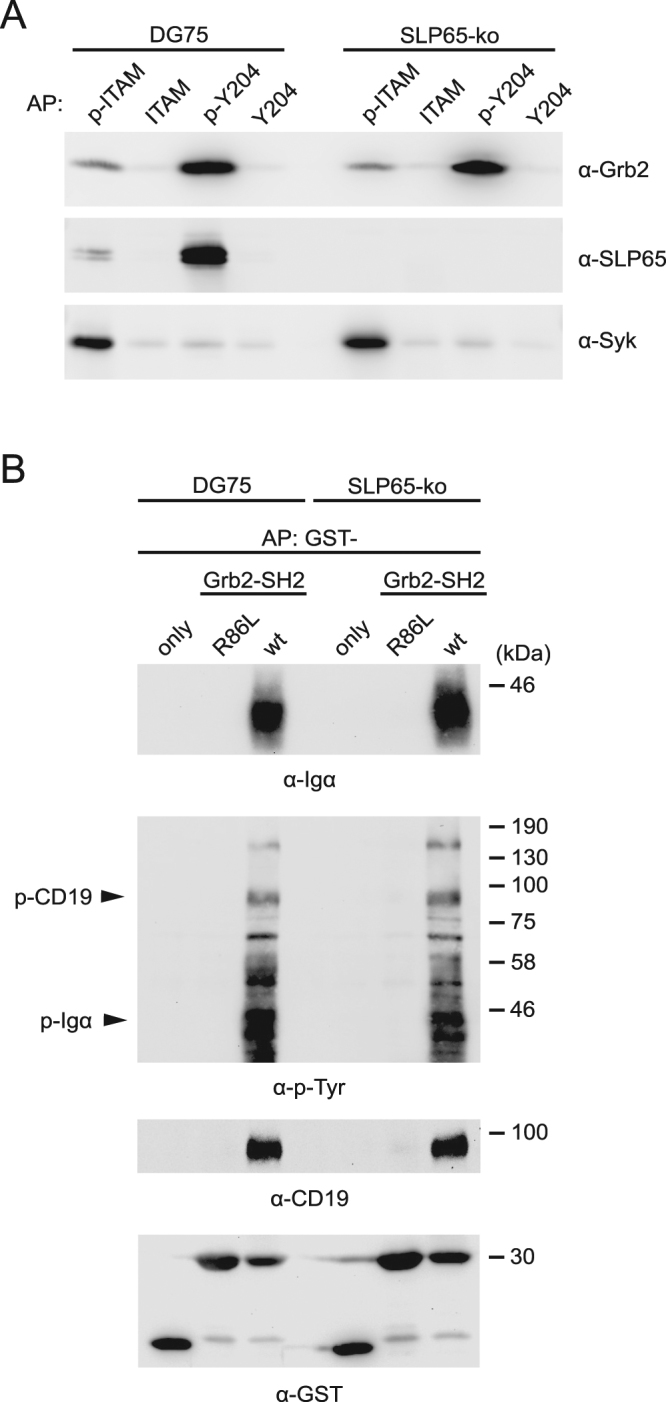
Grb2 interacts directly with the BCR. (**A**) Cleared cellular lysates of parental DG75 B cells and SLP65-deficient cells were used for affinity purifications (AP) using phosphorylated (p-) and non-phosphorylated peptides containing either the ITAM or the non-ITAM (Y204) tyrosine phosphorylation motifs of Igα. In p-ITAM both ITAM tyrosine residues are phosphorylated, whereas in p-Y204 only the non-ITAM tyrosine residue at position 204 of Igα is phosphorylated. The N-terminally biotinylated peptides and their associated binding partners were purified with streptavidin sepharose, followed by immunoblot analysis using the indicated antibodies. (**B**) The same cells as in (A) were stimulated via their endogenous mIgM-BCRs for three minutes and cleared cellular lysates were used for affinity purifications using either the native Grb2 SH2 domain (wt) or an inactivated variant (R86L) coupled to GST. GST without SH2 domain (only) served as an additional control. Bound proteins were analyzed by immunoblotting using the indicated antibodies.

Since inactivation of the SH2 domain of Grb2 abolished both, its Ca^2+^-amplifying function downstream of the mIgE-ITT as well as its capacity to activate the Erk MAP kinase cascade on stimulation of ITT-containing and ITT-less BCR isotypes, we sought to identify binding partners for this domain in BCR-activated B cells. Since the interaction between Grb2 and the mIgE ITT-motif was already established (see Supplementary Figs 5 and 6), we used lysates of DG75 cells that were activated via their endogenous mIgM-BCR as source to isolate interaction partners of the Grb2 SH2 domain. Again, we used the SILAC method to quantitatively identify affinity-purified proteins using mass spectrometry (Supplementary Fig. 21). Among the identified proteins were some that had previously been described as binding partners for the Grb2 SH2 domain such as Dok3, CD22, SHIP and Gab1. Surprisingly, among the proteins showing the strongest enrichment were several components of the BCR complex, including Igα and the immunoglobulin heavy and light chains of the endogenous mIgM-BCR of DG75 B cells (Supplementary Fig. 21). This finding suggested that the BCR itself may recruit Grb2 to its phosphorylated ITAM or non-ITAM tyrosine residues, very much like receptor tyrosine kinases do. To test this scenario in more detail, we used phosphorylated peptides encompassing the ITAM and non-ITAM phosphorylation motifs of Igα to affinity purify proteins from lysates of human B cells (Fig. 8A and Supplementary Fig. 22A). Indeed, Grb2 was purified with the Igα phospho-peptide containing the non-ITAM binding motif including tyrosine 204 (Y204). However, this binding motif is known to interact with SLP65, which in turn is a constitutive ligand for Grb2. Hence, to test whether the interaction between Igα-Y204 and Grb2 is direct or indirect (via SLP65), we repeated the experiment using the SLP65-deficient DG75 cells. The interaction between Igα-Y204 and Grb2 was not reduced in the absence of SLP65 (Fig. 8A), suggesting that there is a direct association of Grb2 and the BCR via the non-ITAM Y204 phospho-tyrosine motif in Igα. To further test this interaction, we used the Grb2 SH2 domain fused to GST for affinity purifications from lysates of parental DG75 B cells and the SLP65-deficient subline. Immunoblot analysis of the purified proteins verified the SLP65-independent interaction of Grb2 and Igα as well as several other tyrosine-phosphorylated proteins, including the B cell co-receptor CD19 (which was not identified in the mass spectrometric approach) (Fig. 8B and Supplementary Fig. 22B). Together, these data show that Grb2 may use various ways to get recruited to the plasma membrane following BCR stimulation, since it can bind with its SH2 domain to a variety of transmembrane or membrane-associated signaling proteins in B cells, one of which is the Igα subunit of the BCR itself.

## Discussion

The adaptor protein Grb2 was initially described to serve a negative regulatory function in the BCR signaling pathway since it attenuated Ca^2+^ mobilization in the chicken B cell line DT40 as well as in mouse B cells^16–18^. However, this categorization turned out to be too simplistic since Grb2 also acts as a signal amplifier when recruited to ITT motifs of memory-type BCR isotypes^12,13,40^. This signal amplification is essential for reactivation of IgG-switched memory B cells in secondary antibody responses^14^. Here we showed that mIgE-BCRs recruit Grb2 and the related adaptor protein GRAP to their ITT motifs to amplify canonical BCR signaling. This signal amplification enhances ITAM-induced Ca^2+^ mobilization as well as activation of the Erk MAP kinase cascade and might improve activation of IgE-switched cells *in vivo*. Another mechanism by which the activation of IgE-switched B cells is promoted *in vivo* involves a short region in the extracellular part of mouse mIgE, referred to as extracellular membrane-proximal domain (EMPD), which associates with the B cell co-receptor CD19^41^. This association seems to cause an antigen-independent activation of mIgE-BCR and CD19 signaling, which together results in a rapid and efficient differentiation of mIgE-switched post germinal center B cells into short-lived IgE-secreting plasma cells^41,42^. However, when analyzing human mIgE-BCRs, we did not find signs of autonomous, i.e. antigen-independent signaling (this study and^43^). This may be due to the low sequence conservation between the EMPDs of human and murine mIgE^43^, which might prevent the association between the human orthologues of mIgE and CD19. Yet, human and murine mIgE orthologues share a functional ITT signaling motif that amplifies ITAM signals by recruiting Grb2 family adaptor proteins.

However, the positive role of Grb2 is not restricted to signaling of ITT-containing BCR isotypes, since also ITT-less mIgM-BCRs employ Grb2 family adaptor proteins to couple ITAM-induced signals to activation of the Ras/Erk MAP kinase pathway. This coupling of BCR activation to Ras and Erk thus far has been primarily studied in mouse B cells and the chicken B cell line DT40. In these model systems, Erk activation is critically dependent on the activity of PLCɣ and the subsequent activation of DAG-responsive signaling proteins including members of the protein kinase C (PKC) family and RasGRP isoforms^33–35^. In human B cells a similar mechanism was proposed based on the analysis of RasGRP3 and PKC in the Burkitt lymphoma line Ramos^44,45^. However, primary human B cells were not investigated in these studies. Like the DG75 B cell line, primary human B cells express RasGRP2, which however is not capable of connecting BCR engagement to activation of the Ras/Erk MAP kinase pathway. RasGRP2 possesses an unusual C1 domain that, unlike those of RasGRP1 and RasGRP3, does not bind DAG and hence is not regulated by the activity of PLCɣ^46,47^. Whether this lack of DAG responsiveness is responsible for the failure of RasGRP2 to connect BCR engagement to activation of the Erk MAP kinase pathway remains to be investigated. In mouse B cells there is a striking separation of RasGRP isoforms between B1 and B2 B cells. Whereas RasGRP1 is highly expressed in B1 cells, RasGRP3 is exclusively found in B2 cells. Accordingly, RasGRP1-deficient mice have low B1 cell numbers and reduced natural serum IgM titers^48^. However, even combined deficiency of RasGRP1 and RasGRP3 has no detectable effect on overall B cell development, despite a severely impaired activation of the Erk MAP kinase pathway in mature mouse B cells^33^. Given the well documented importance of Ras and Erk for B cell development^22–24^, this finding implicates that developing B cells in the mouse must have alternative mechanisms to link (pre-) BCR signaling to activation of Ras and Erk. Our results suggest that this alternative pathway may be mediated by Grb2/GRAP and Sos. However, B cell-specific deletion of Grb2 in the mouse has only subtle effects on B cell development in the bone marrow, yet it causes a drastic reduction in the total number of mature B cells^16,49^. Whether developing B cells express and hence use GRAP or RasGRPs to compensate for the loss of Grb2 remains unclear.

Previous studies have suggested an interplay of RasGRPs and Grb2/Sos to activate the Erk MAP kinase pathway in lymphocytes^50–52^. However, these studies were primarily done in T cells and the chicken B cell line DT40 that indeed co-expresses both classes of Ras activators, Grb2/Sos and RasGRPs. Since primary human B cells do hardly express PLCɣ-regulated RasGRP isoforms and, unlike in DT40 cells, can efficiently activate the Erk pathway in the absence of PLCɣ activity, it appears questionable whether the proposed interplay of RasGRPs and Grb2/Sos is operating in human B cells.

The mechanism by which Grb2 family adaptors are recruited to the activated BCR or its immediate environment is not completely understood but most likely involves an SH2 domain-mediated interaction with a plasma-membrane associated phospho-protein. In a proteomics-based approach we previously identified interaction partners of Grb2 in mouse B cells^53^. Here, we extended these studies and specifically identified binding partners of the Grb2 SH2 domain in human B cells. This approach revealed that Grb2 can bind to several B cell transmembrane proteins including CD19, CD22 and also the ITT and Igα, i.e. the BCR itself. If Grb2 (and GRAP) prefer one of those interaction partners to connect BCR stimulation to activation of Ras and Erk or if the Grb2 SH2 domain binding partners are functionally interchangeable with regard to activation of Erk remains to be investigated. However, out results indicate that the BCR itself is a membrane recruitment anchor for Grb2/Sos and thus functionally mimics receptor tyrosine kinases like the EGF receptor. Notably, Grb2 binds to the BCR either via the ITT motif or – in ITT-less BCR isotypes – primarily through an interaction with the non-ITAM tyrosine phosphorylation motif Y204 in Igα. This interaction motif was previously shown to recruit further adaptor proteins, such as SLP65 and Nck and by doing so to facilitate BCR-induced Ca^2+^ mobilization and activation of PI3 kinase, respectively^8,9,54^. Thus, whereas the ITAMs of the BCR are potent activators of cytoplasmic protein tyrosine kinases, Igα Y204 and the ITT motifs of memory-type BCRs seem to have specialized in recruiting catalytically inert adaptor proteins to facilitate the activation of downstream signaling pathways.

In summary our results show that in human B cells the adaptor proteins Grb2 and GRAP serve a dual function: first to amplify signaling by mIgE-containing BCRs, which enhances their sensitivity for antigen and second to connect BCR stimulation to activation of the mitogenic Ras/Erk MAP kinase pathway. Thus, in human B cells these adaptor proteins have critical functions in B cell activation and do not represent mere inhibitory adaptor proteins as previously suggested. Also, our findings reveal that results obtained in non-human lymphocyte model systems should be carefully evaluated in human cells, which will be greatly facilitated by the recent developments in the field of genome editing.

## Methods

### Cells and TALEN-mediated gene targeting

The human Burkitt lymphoma B cell lines DG75 and Ramos were purchased from the German Collection of Microorganisms and Cell Cultures (DSMZ, Braunschweig, Germany). The DG75EB variant of the cells, which expresses the murine cationic amino acid transporter 1 (SLC7A1) to make them susceptible to infection with MMLV-based retrovirus particles, and the Grb2-deficient subline of DG75 cells were described before^13^. DG75 cells deficient for SLP65 or Grb2 and GRAP or PLCɣ1 and PLCɣ2 were generated by TALEN-mediated genome editing. TALEN cassettes were designed using the TAL Effector Nucleotide Targeter 2.0 (https://tale-nt.cac.cornell.edu/) and generated using a modified version of the ‘Golden Gate’ TALEN assembly method^55^. The plasmid kit used for generation of TALENs was a gift from Daniel Voytas and Adam Bogdanove (Addgene kit # 1000000024). Modifications included shortening of the linker regions N- and C-terminal of the DNA binding modules and optimization of the FokI nuclease by introducing ‘Sharkey’ mutations that cause a higher activity without increasing off-target effects^56^. Furthermore, we introduced an N-terminal HA-tag and an improved Kozak translation initiation sequence. The resulting modified TALEN backbone vector was named pTAL4Titanium. The two different TALEN constructs for each mutagenesis approach (designated left and right TALEN, respectively) were cloned into expression vectors pmax-IE and pmax-IR, containing an IRES-EGFP (IE) and an IRES-tagRFP (IR) cassette, respectively. DG75 cells were transiently transfected with both plasmids using the Amaxa Nucleofector™ II device (Lonza) in combination with the Lonza Human B cell Nucleofector™ Kit (program T-015). Two days after electroporation, EGFP/RFP double-positive cells were sorted, expanded, subcloned and screened for deficiency of the target gene (see supplementary figures for further details). All cells were maintained in RPMI1640 + Glutamaxx (Biochrom) supplemented with 10% heat-inactivated FCS and antibiotics.

### Expression vectors, antibodies and peptides

The cDNA encoding the long isoform of the human membrane-bound ε immunoglobulin heavy chain (εm), PCR-amplified from the IgE-secreting myeloma line U266, was a gift of Dr. Jürgen Frey, Bielefeld, Germany. The V region of that cDNA was replaced by the NP-specific B1–8 region containing an intron via an endogenous PshAI restriction site. To generate the short εm isoform, 52 codons of the EMPD were deleted by mutagenesis PCR. Mutations of the ITT motif were introduced by the same method. All immunoglobulin heavy chains were expressed using the pMSCV retroviral vector (Clontech). Mouse Grb2 and human GRAP cDNAs were expressed using the retroviral vectors pMIGRII (containing an IRES-driven EGFP cassette) or pMIRFP in which the EGFP-cDNA from MIGRII was replaced by a cDNA encoding tag-RFP, respectively. Mutant variants of Grb2 or GRAP carrying inactivating amino acid substitutions in either the N-terminal SH3 domain (W36K), the SH2 domain (R86K), or C-terminal SH3 domain (W193K for Grb2 and W195K for GRAP) were generated using site directed mutagenesis and cloned into the pMIGRII or pMIRFP vectors for expression. GST fusion proteins containing variants of the Grb2 or GRAP SH2 domains were expressed in *E. coli* using the pGEX4T-1 vector. The biotinylated human-ε-phospho-ITT peptide (Biotin-RPQTSLD(p)YTNVLQPHA) was synthesized by CASLO ApS (Lyngby, Denmark). Igα-derived (phospho-) peptides were synthesized by Eurogentec (Liège, Belgium). Antibodies against RasGRP1 (clone A7), RasGRP3 (B10), PLCɣ1 and PLCɣ2 (both polyclonal) were from Santa Cruz. The antibody against RasGRP2 (rabbit polyclonal) was from Abcam. Antibodies to phospho-Erk (E10), phospho-tyrosine (100), phospho-Mek1 (Ser 298), Mek1/2 (L38C12), β-Actin (13E5) and CD19 (polyclonal) were from Cell Signaling Technology. The pan-Erk antibody (clone 16, raised against Erk2) was from BD Biosciences. The anti-Grb2 antibody (3F2) was from Millipore, the anti-SLP65 antibody (2C9) from BAbCo, the polyclonal anti-GRAP antibody (raised against the C-terminus of GRAP) from Atlas Antibodies and the anti-GST antibody from MoBiTec. Polyclonal goat anti-human ε Ig heavy chain antibodies and anti-goat-IgG (H&L)-Alexa Fluor 647 F(ab’)_2_ fragments were from Abcam. Anti-human IgM F(ab’)_2_ and anti-mouse IgG F(ab’)_2_ fragments were from Jackson ImmunoResearch or SouthernBiotech. HRPO-coupled secondary antibodies for immunoblotting were from SouthernBiotech. Anti-CD19-Brilliant Violet 421 (HIB19) from Biolegend, goat anti-human IgM-FITC F(ab’)_2_ from SouthernBiotech and anti-pErk-Alexa Fluor® 647 (20 A) from BD Biosciences were used to stain fixed and permeabilized primary human B cells.

### Retroviral transduction of B cells

Retroviral transduction was done using the Plat-E retroviral packaging cell line. Two days after transfection, the virus-containing supernatant was used to infect B cells. If applicable positively transduced cells were selected using 3 µg/ml puromycin (InvivoGen). Alternatively, cells that were transduced using either the pMIGRII or pMIRFP vectors were sorted for expression of either EGFP or tagRFP at the Cell Sorting Facility of the University Medical Center Goettingen.

### Biochemical assays

For BCR stimulation, cells were incubated with 20 µg/ml goat anti-human IgE antibodies or goat F(ab’)_2_ fragments against human IgM at 37 °C in RPMI for the indicated times. Preparation of cellular lysates in 1% NP40-containing lysis buffer for affinity purifications and western blot analyses was done as described^8^. Briefly, the cells were lysed with lysis buffer composed of 50 mM Tris-HCl (pH 7.8), 137 mM NaCl, 0.5 mM EDTA, 1 mM sodium orthovanadate, 10% (w/v) glycerol, 1% (w/v) NP40 and a protease inhibitor cocktail (Sigma Aldrich, P2714). Lysis was performed on ice for 10 min and the cell debris was pelleted at 20,000 × g at 4 °C for 10 min. The supernatant containing the cell lysate was mixed with reducing SDS-PAGE sample buffer, boiled at 95 °C for 5 min and analyzed by SDS-PAGE and immunoblotting. For affinity purification of proteins from B cell lysates using biotinylated peptides or GST fusion proteins, cell lysates were prepared as described above from 3 × 10^7^ DG75EB cells and incubated with 2 µM peptides or 20 µg GST fusion proteins bound to glutathione-sepharose. Biotinylated peptides and associated binding partners were purified using streptavidin-sepharose beads under gentle rotation at 4 °C for 2 hours. The samples were repeatedly washed in lysis buffer, before they were subjected to SDS-PAGE and immunoblotting. For mass spectrometric analyses of purified proteins, 1.5 × 10^8^ cells were used that were grown in either standard medium or medium that contained “heavy” labelled amino acids, i.e. 0.115 mM ^13^C_6_^15^N_4_ L-arginine and 0.275 mM ^13^C_6_^15^N_2_ L-lysine (SILAC method). Mass spectrometric identification of purified proteins was done at the Proteomics Core Facility of the University Medical Center Goettingen by tandem mass spectrometry-MS/MS. Data were analyzed using Microsoft Excel and Perseus 1.5.5.0 software^57^. Immunoblot images were processed using Photoshop CS4 and Corel Draw software. Quantification of band intensities was done using Image J and subsequent analysis in Microsoft Excel. Band intensities were normalized by setting the highest value to 1.0 and calculating the other values accordingly.

### Isolation of primary human B cells

Primary human B cells from peripheral blood of healthy donors were isolated by magnetic cell sorting using the B cell isolation kit II (Myltenyi Biotec, Bergisch Gladbach, Germany) according to the manufacturer´s instructions. The purity of the isolated B cells was tested by surface staining of CD19 and IgM and was typically around 95% or higher.

Experiments involving human participants were approved by the ethical review committee of the University Medical Center Goettingen and were performed in accordance with relevant guidelines and regulations. An informed consent was obtained from all participants.

### Measurement of intracellular free Ca^2+^

For analyzing intracellular Ca^2+^ mobilization kinetics, 1 × 10^6^ cells per measurement were incubated with 1 µM Indo-1-AM (Molecular Probes) and 0.015% Pluronic-F-127 in RPMI containing 10% FCS at 30 °C with mild agitation. The cells were then washed and resuspended in Krebs Ringer solution, which was composed of 10 mM HEPES (pH 7.0), 140 mM NaCl, 4 mM KCl, 1 mM MgCl_2_, 1 mM CaCl_2_ and 10 mM glucose. Basal Ca^2+^ levels were measured for 30 seconds after which the samples were stimulated with the indicated reagents. Measurements were done at the BD LSRII and data analysis was done using FlowJo (FlowJo LLC) and Microsoft Excel. Primary human B cells purified from blood of healthy donors were prepared as described above and stimulated with 15 µg/ml goat anti-human IgM F(ab’)_2_ (SouthernBiotech). The PLC inhibitor U73122 (Tocris Bioscience) was added to the cells at the indicated concentrations 1 minute before BCR stimulation.

### Intracellular staining of phosphorylated Erk

Prior to stimulation of the BCR, cells were incubated in FCS-free RPMI1 °C640 for 30 minutes at 37 . Subsequently 0.5 × 10^6^ cells were stimulated with 10 µg/ml goat anti-human IgM F(ab’)_2_ fragments for the indicated times. The stimulation was stopped by adding an equal amount of Cytofix buffer (BD Biosciences). Permeabilization and staining of the cells was done in Phos-flow Perm/Wash buffer I (BD Biosciences). The Alexa-Fluor 647-coupled anti-phospho-Erk1/2 (pT202/pY204) antibody (clone 20 A) was from BD Biosciences. The mean fluorescence intensity (MFI) for each sample was normalized to maximal Erk activation in each experiment, which was defined as 1.0. For analysis of Erk phosphorylation in primary human B cells, peripheral blood mononuclear cells (PBMCs) were isolated from blood of healthy donors. Blood was mixed 1:1 with PBS containing 1 mM EDTA and layered on LeucoSep™ tubes (Greiner Bio-One) with Roti®-Sep (Roth) and centrifuged at 800 × g at room temperature for 20 min. The PBMC layer was collected and washed in PBS containing 1 mM EDTA and then rested in RPMI containing 10% FCS for 2 hours at 37 °C. Thereafter, 0.5 × 10^6^ cells were either left unstimulated or stimulated with 15 µg/ml goat anti-human IgM F(ab’)_2_ (SouthernBiotech) in the absence or presence of 4 μM PLC inhibitor U73122 (Tocris Bioscience), followed by BCR stimulation at 37 °C for 5 min. The reaction was stopped by adding an equal amount of BD Cytofix™ and the cells were fixed at 37 °C for 10 min with mild agitation. The cells were harvested by centrifugation at 300 × g at room temperature for 5 min. Permeabilization was done on ice for 30 min with Perm Buffer III (BD Biosciences) and cells were stained in PBS containing 0.5% BSA with the respective antibodies for 40 min. The cells were then washed and measured on the BD LSR II and data analysis was done using FlowJo (FlowJo LLC) and Microsoft Excel. The mean fluorescence intensity of phosphorylated Erk for the unstimulated sample was normalized to 1.0 and all other values were calculated accordingly.

### Data availability

The datasets generated and analyzed during the current study (e.g. proteomic analyses) as well as reagents and cell lines that were generated and used are available from the corresponding author on reasonable request.

## Electronic supplementary material

Supplementary Information
