# Supplementary material for: Grb2 and GRAP connect the B cell antigen receptor to Erk MAP kinase activation in human B cells
Source: Sci Rep. 2018 Mar 9;8:4244. doi: 10.1038/s41598-018-22544-x (PMC5844867; doi:10.1038/s41598-018-22544-x)
Supplement: Supplementary file 1 — Supplementary Information [file 41598_2018_22544_MOESM1_ESM.pdf]

Grb2 and GRAP connect the B cell antigen receptor to Erk MAP kinase activation in human B cells

by Kanika Vanshylla, Caren Bartsch, Christoffer Hitzing, Laura Krümpelmann, Jürgen Wienands and Niklas Engels

Supplementary Figures

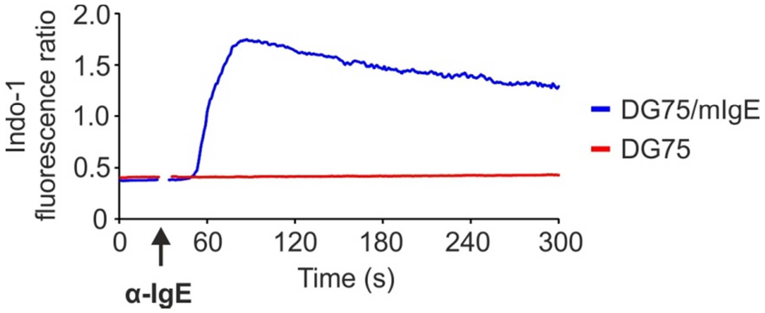

Supplementary Figure 1 | The stimulating anti-IgE antibody does not activate mIgM-BCRs. Ca^{2+} mobilization kinetics of DG75 cells expressing an endogenous mIgM-BCR (red curve) or expressing in addition the short isoform of human mIgE (blue curve). After recording baseline Ca^{2+} levels for 30 seconds, cells were treated with 10 µg/ml anti-IgE antibodies (indicated by arrow).

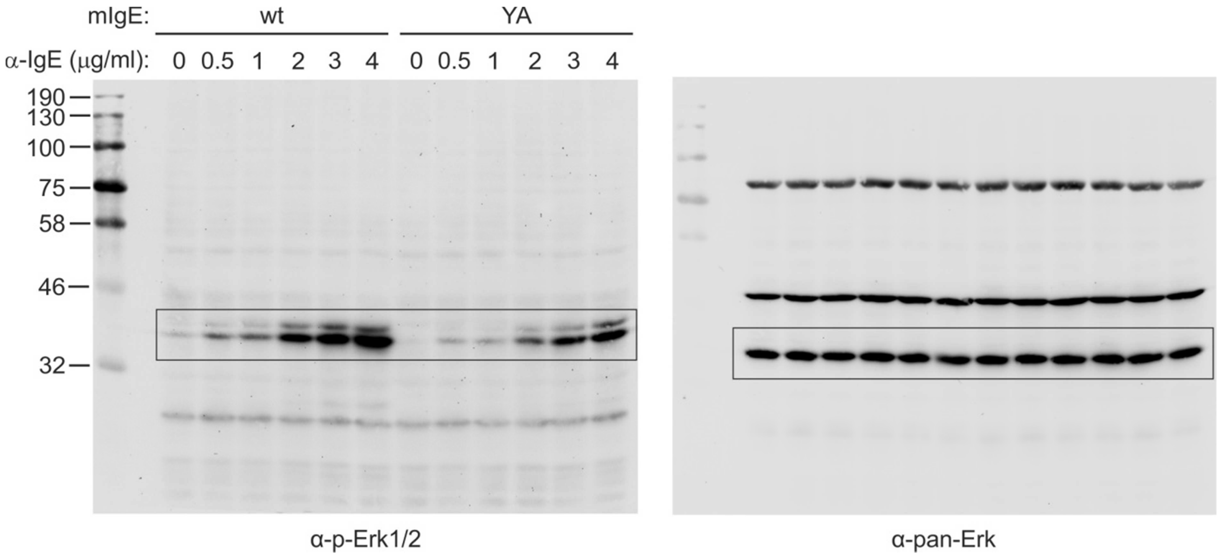

Supplementary Figure 2 | Uncropped western blots shown in Figure 1E. Nitrocellulose membrane was sequentially developed from left to right. The cropped parts are indicated by boxes.

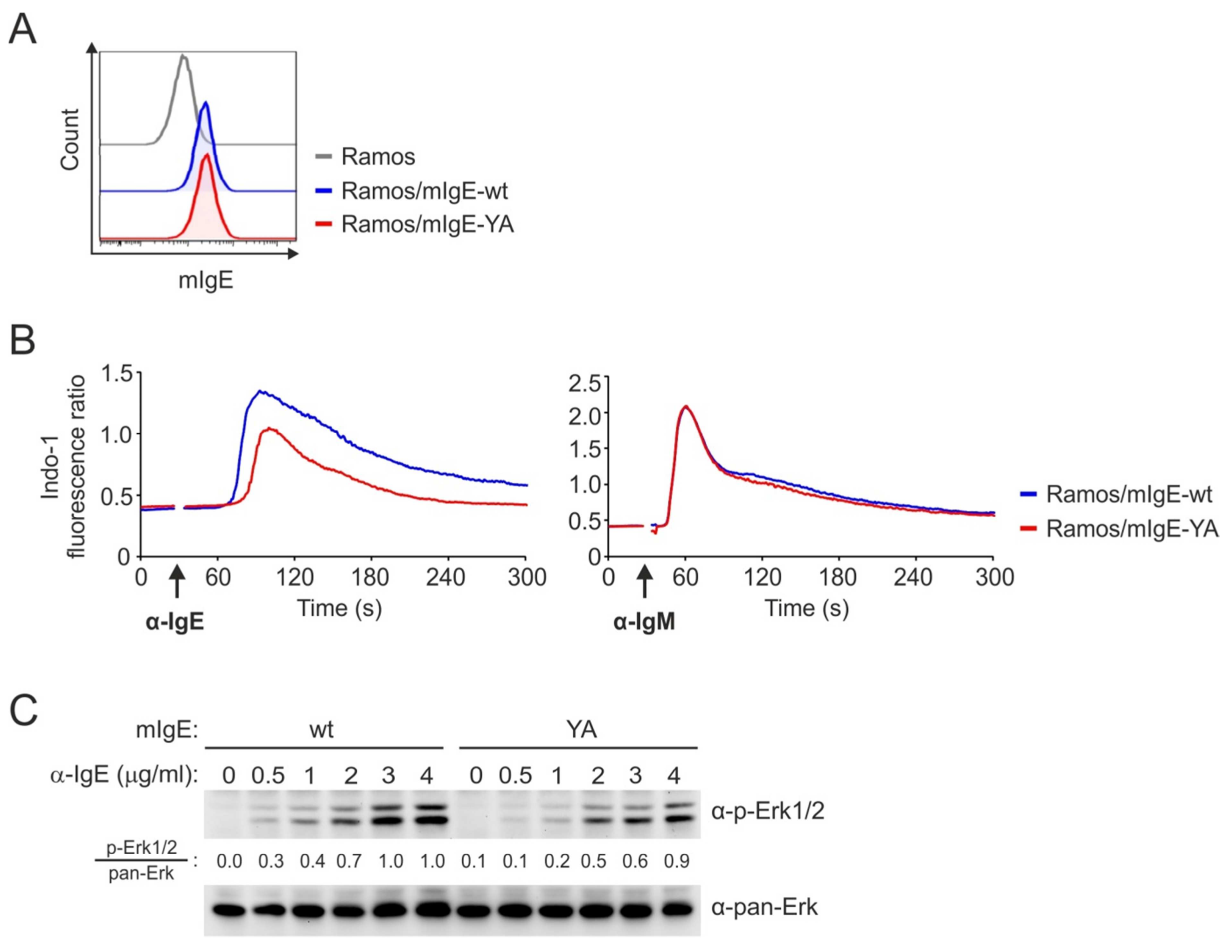

Supplementary Figure 3 | Analysis of mIgE-BCR signaling in Ramos B cells. (A) Ramos B cells were retrovirally transduced to express either the wild type short isoform of human mIgE (mIgE-wt, blue curve) or a ITT tyrosine to alanine mutant variant (mIgE-YA, red curve). Surface expression of the different mIgE variants was analyzed by flow cytometry. Parental Ramos cells are shown as control (grey curve). (B) BCR-induced Ca^{2+} mobilization in the cells shown in (A) was analyzed on stimulation of the cells with either 10 µg/ml anti-IgE antibodies (left graph) or with 20 µg/ml anti-IgM $F(ab')_2$ fragments as control (right graph). (C) Activation of Erk kinases following stimulation of wild type or ITT-mutant (YA) mIgE-BCRs was analyzed by immunoblotting of cleared cellular lysates using antibodies to phospho-Erk (α-p-Erk1/2) and non-phosphorylated Erk (α-pan-Erk). Band intensities were quantified and the ratio of signal intensities for phospho-Erk divided by the signal intensities for total Erk is given. Maximal Erk activation was set to 1.0 and all other signal intensities were calculated accordingly. Data are representative of three independent experiments.

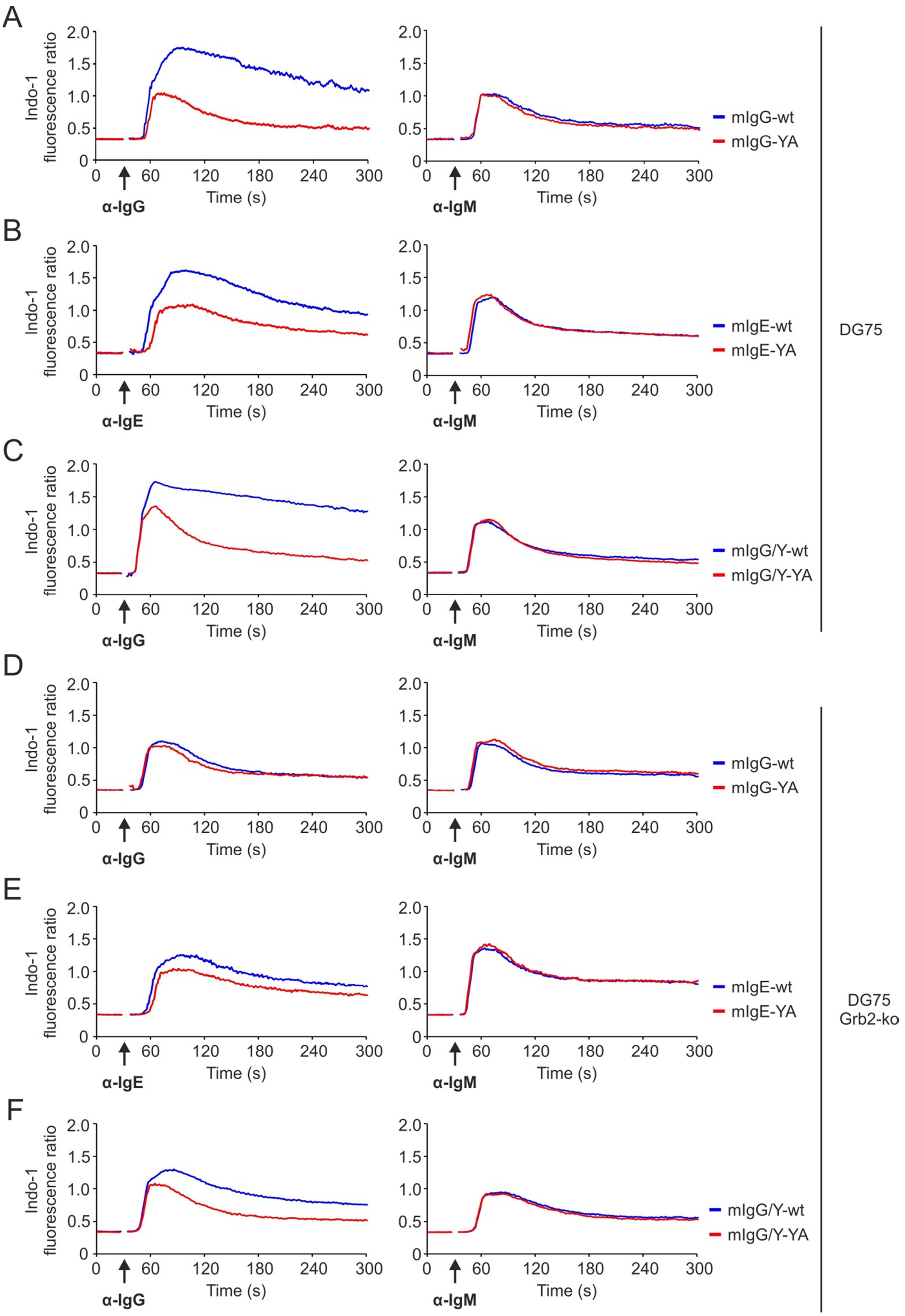
A
B
C
D
E
F
Indo-1 fluorescence ratio
Time (s)
α-IgG
α-IgE
α-IgM
mIgG-wt
mIgG-YA
mIgE-wt
mIgE-YA
mIgG/Y-wt
mIgG/Y-YA
DG75
DG75 Grb2-ko

Supplementary Figure 4 | The ITT motifs of mIgE and mIgY are partially independent of Grb2. (A) Wild-type DG75 B cells expressing mIgG-BCR were stimulated with either anti-IgG antibodies (left graph) or anti-IgM F(ab')$_2$ fragments as control (right graph). (B, C) DG75 cells expressing either human mIgE-BCRs (B) or a chimeric membrane-bound mIgG/Y consisting of the extracellular and transmembrane regions of mouse ɣ2am and the cytoplasmic tail of reptilian (*Anolis carolinensis*) υm (C) were analyzed accordingly. (D-F) The same mIg constructs as in (A-C) were expressed in Grb2-deficient DG75 B cells (Grb2-ko) and analyzed the same way.

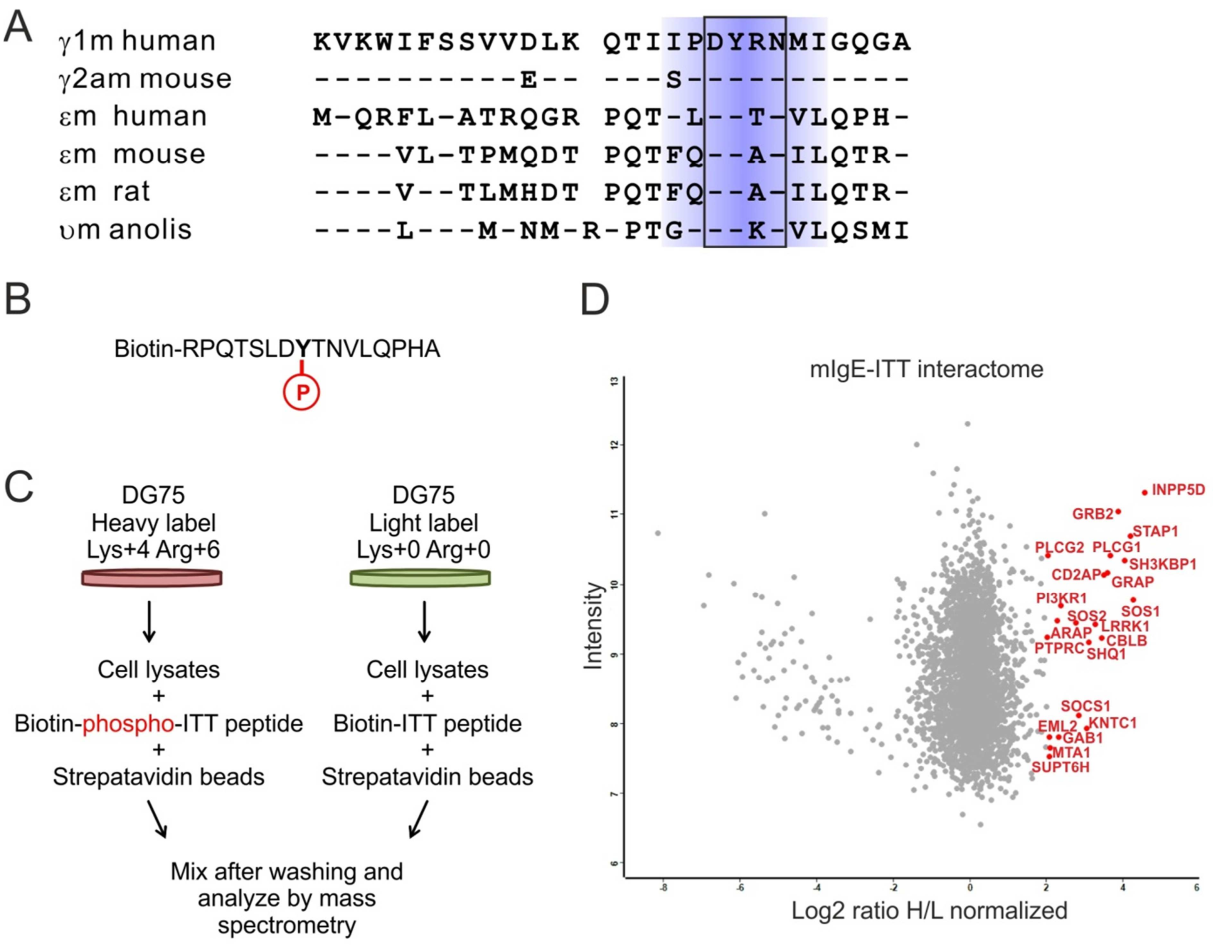

E

Ratio H/L normalized	No. Peptides	Gene name	Protein ID	Protein name
4.5656	99	INPP5D	Q92835	Phosphatidylinositol 3.4.5-trisphosphate 5-phosphatase 1
4.25626	63	SOS1	Q07889	Son of sevenless homolog 1
4.19077	30	STAP1	Q9ULZ2	Signal-transducing adaptor protein 1
4.02751	39	SH3KBP1	Q96B97	SH3 domain-containing kinase-binding protein 1
3.86898	23	GRB2	P62993	Growth factor receptor-bound protein 2
3.65432	65	PLCG1	P19174-2	1-phosphatidylinositol 4.5-bisphosphate phosphodiesterase gamma-1
3.5852	17	GRAP	Q13588	GRB2-related adapter protein
3.498	37	CD2AP	Q9Y5K6	CD2-associated protein
3.44042	24	CBLB	Q13191	E3 ubiquitin-protein ligase CBL-B
3.26881	48	LRRK1	Q38SD2	Leucine-rich repeat serine/threonine-protein kinase 1
3.09667	2	SHQ1	Q6PI26	Protein SHQ1 homolog
3.04429	5	KNTC1	P50748	Kinetochore-associated protein 1
2.83094	4	SOCS1	O15524	Suppressor of cytokine signaling 1
2.75931	58	SOS2	Q07890	Son of sevenless homolog 2
2.36972	34	PIK3R1	P27986	Phosphatidylinositol 3-kinase regulatory subunit alpha
2.30457	5	GAB1	Q13480	GRB2-associated-binding protein 1
2.27569	46	ARAP1	Q96P48	Arf-GAP with Rho-GAP domain. ANK repeat and PH domain-containing protein 1
2.07953	5	MTA1	Q13330	Metastasis-associated protein MTA1
2.07413	3	EML2	O95834	Echinoderm microtubule-associated protein-like 2
2.06516	3	SUPT6H	Q7KZ85	Transcription elongation factor SPT6
2.02751	68	PLCG2	P16885	1-phosphatidylinositol 4.5-bisphosphate phosphodiesterase gamma-2
2.00931	26	PTPRC	P08575	Receptor-type tyrosine-protein phosphatase C

Supplementary Figure 5 | Identification of interaction partners of the human mIgE-ITT motif. (A) Amino acid sequence alignment of different membrane-bound immunoglobulin tail sequences. Dashes indicate amino acid residues that are identical to the human ɣ1m tail; box outlines the ITT core (DYxN) motif; blue background indicates amino acid residues that (may) influence the binding of SH2 domain-containing effector proteins. (B) Amino acid sequence of the (phospho-) peptides used for affinity purification of mIgE-ITT binding partners from lysates of human B cells. (C) Schematic outline of the SILAC (stable isotope labeling of amino acids in cell culture) method. DG75 B cells were grown in either 'light' medium containing standard amino acids or 'heavy' medium containing 0.115 mM $^{13}C_6$ $^{15}N_4$ L-arginine (Arg+6) and 0.275 mM $^{13}C_6$ $^{15}N_2$ L-lysine (Lys+4). Phosphorylated or non-phosphorylated ITT peptides were added to lysates of cells grown in the different media and interaction partners were immobilized by addition of streptavidin sepharose. Identification of bound proteins was done using tandem mass spectrometry-MS/MS. (D) Dot plot of the identified proteins in the mass spectrometric analysis. All identified proteins are plotted according to their 'heavy' versus 'light' ratio of enrichment (H/L) on logarithmic scales. Proteins with H/L ratio > 4 were defined as specific interaction partners of the mIgE-ITT and are indicated in red. (E) List of proteins that were identified as mIgE-ITT interaction partners. Grb2 and GRAP are highlighted in red.

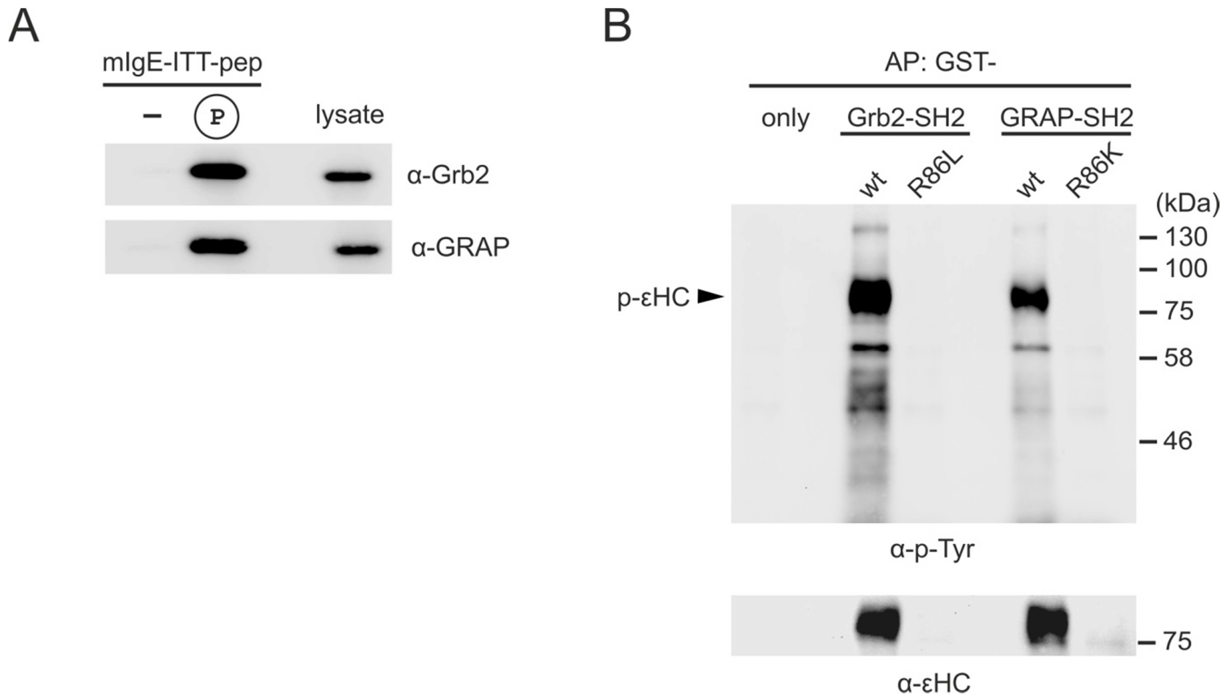

Supplementary Figure 6 | Grb2 and GRAP bind to the mIgE-ITT motif. (A) Affinity purification of mIgE-ITT binding partners using a phosphorylated (P) or non-phosphorylated (-) peptide (see figure S1A) from lysates of DG75 B cells. Binding of Grb2 and GRAP to the peptides was tested by immunoblotting using the indicated antibodies. (B) DG75 B cells were stimulated via their endogenous mIgM-BCRs for three minutes and cleared cellular lysates were used for affinity purifications using either the native SH2 domains of Grb2 or GRAP (wt) or inactivated variants (R86L for Grb2 and R86K for GRAP) coupled to GST. GST without SH2 domain (only) served as an additional control. Bound proteins were analyzed by immunoblotting using the indicated antibodies.

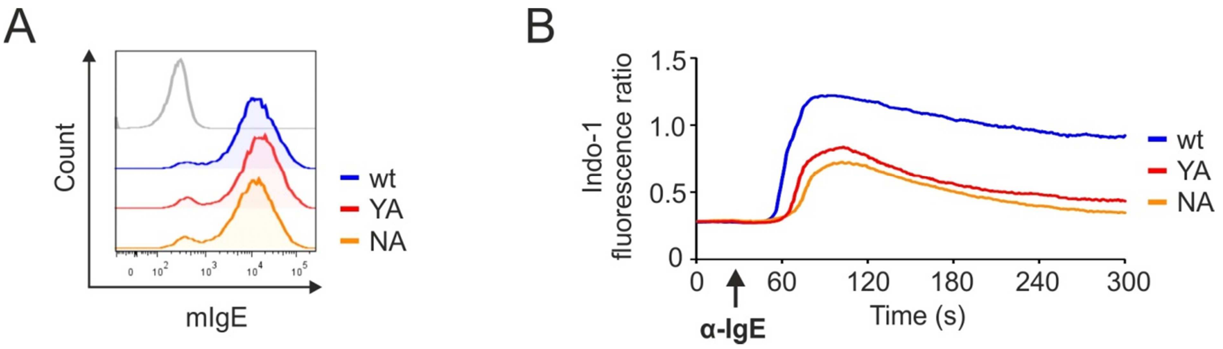

Supplementary Figure 7 | The ability to bind Grb2 and GRAP is essential for the functionality of the mIgE-ITT motif. DG75EB cells were retrovirally transduced to express either wild type (wt), an ITT tyrosine to alanine mutant (YA) or an ITT asparagine to alanine (NA) mutant variant of mIgE. Surface expression of the mIgE variants in sorted cells is shown in (A). (B) BCR-induced mobilization of Ca^{2+} was analyzed on stimulation of the cells with 10 µg/ml anti-IgE antibodies.

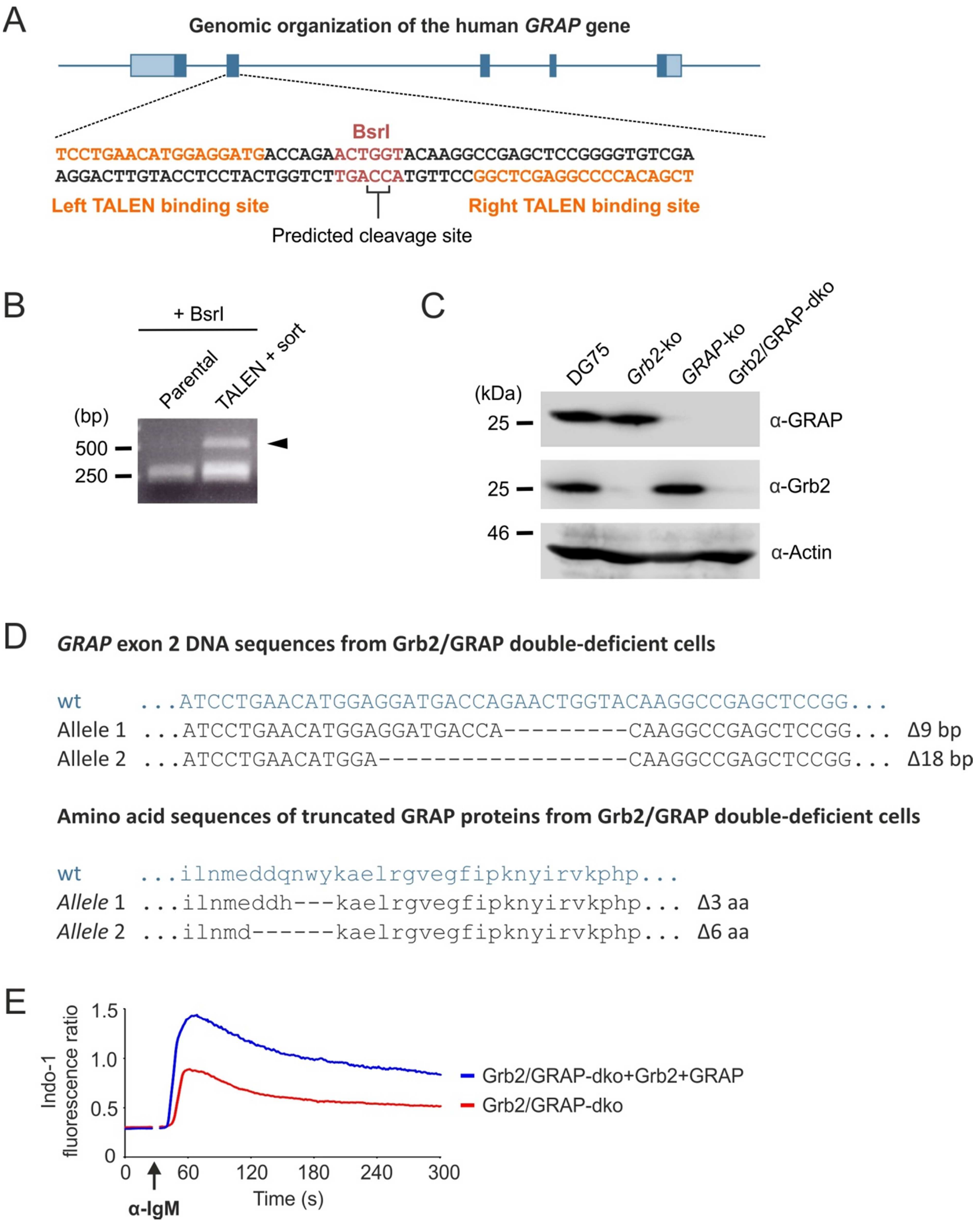

Supplementary Figure 8 | Generation of a Grb2/GRAP double-deficient subline of DG75 B cells. (A) Schematic representation of the locus of the human *GRAP* gene (not drawn to scale). The TALEN constructs were designed to introduce a double-strand break within exon 2. Any indel mutation resulting from erroneous DNA double strand break repair would destroy a BsrI restriction site. Grb2-deficient DG75 cells [1] were used to generate Grb2/GRAP double knock-out (dko) cells. (B) Genomic DNA from sorted cells that had expressed the TALEN constructs for several days (or untreated

parental cells as control) was used to amplify exon 2 of the *GRAP* gene. The amplicon was then cleaved with BsrI to test for TALEN activity. The arrowhead indicates cleavage-resistant exon 2. (C) Immunoblot analysis of lysates from various DG75 variants. (D) DNA sequences showing nucleotide deletions of the mutant *GRAP* alleles and the resulting amino acid sequences of curtailed GRAP proteins. (E) BCR-induced Ca^{2+} mobilization kinetics of Grb2/GRAP double-deficient cells and cells reconstituted with both Grb2 and GRAP on stimulation of the mIgM-BCR.

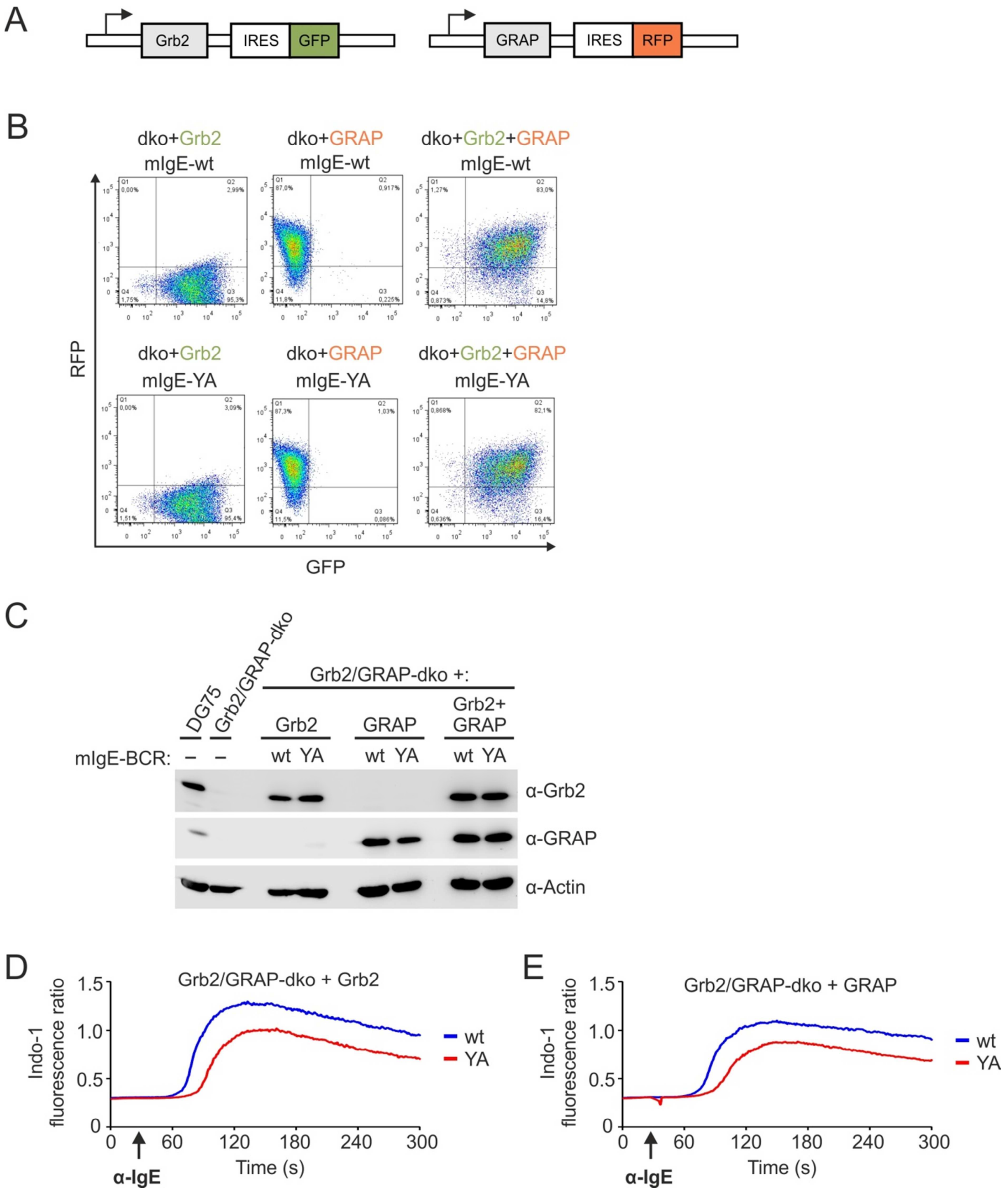

Supplementary Figure 9 | Reconstitution of Grb2/GRAP double-deficient DG75 B cells with Grb2 and GRAP. (A) Retroviral expression vectors for Grb2 and GRAP contained internal ribosomal entry site (IRES) elements for co-expression of EGFP or tagRRP, respectively. (B) Flow cytometric analyses showing expression of EGFP (Grb2) and RFP (GRAP) after cell sorting. (C) Immunoblot analysis of Grb2 and GRAP expression in the indicated cell types. (D & E) BCR-induced Ca^{2+} mobilization kinetics of Grb2/GRAP double-deficient cells reconstituted with either Grb2 (D) or GRAP (E) on stimulation of the mIgE-BCR.

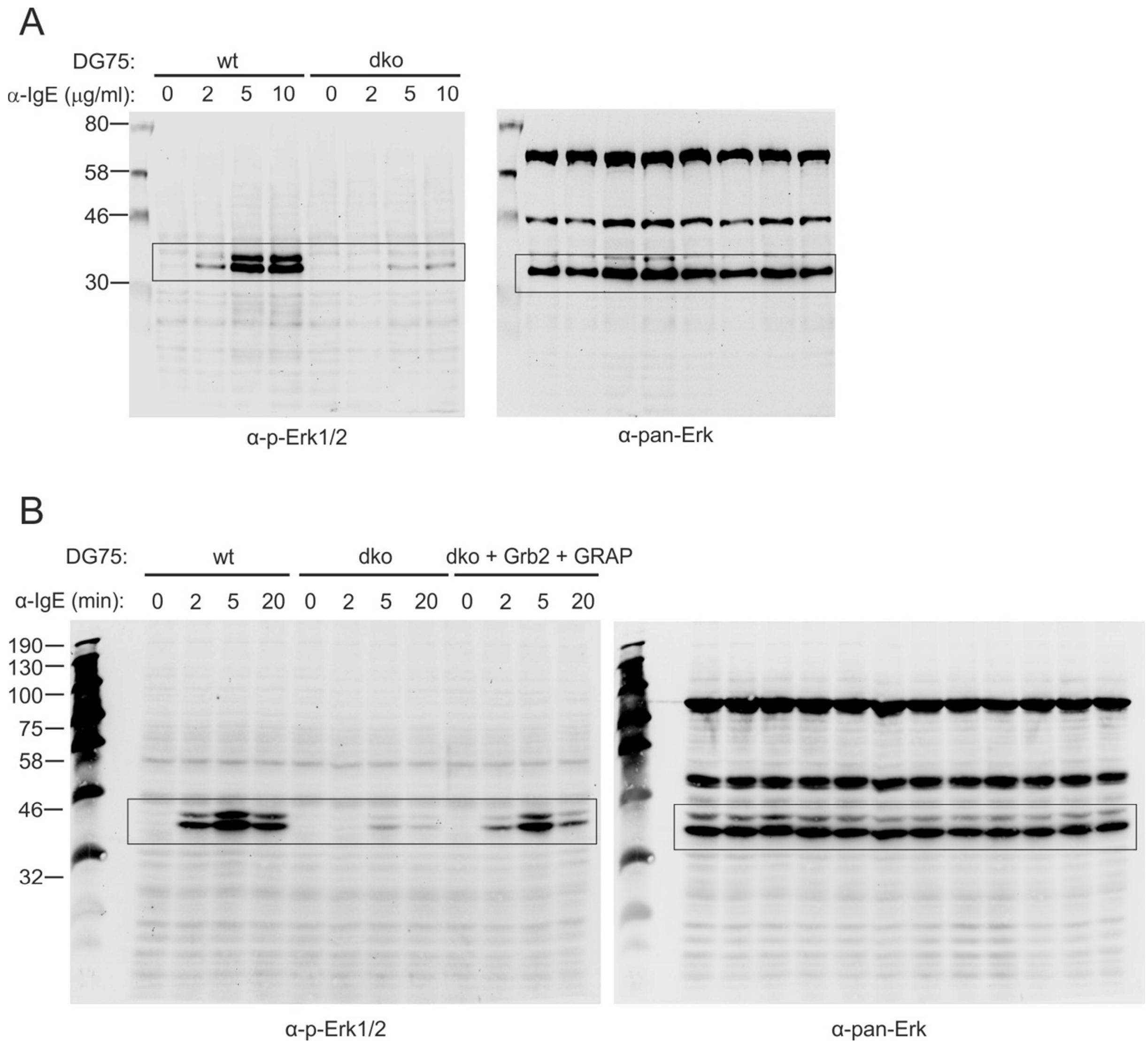

Supplementary Figure 10 | Uncropped western blots shown in Figure 2E (A) and Figure 2F (B). Nitrocellulose membranes were sequentially developed from left to right. The cropped parts are indicated by boxes.

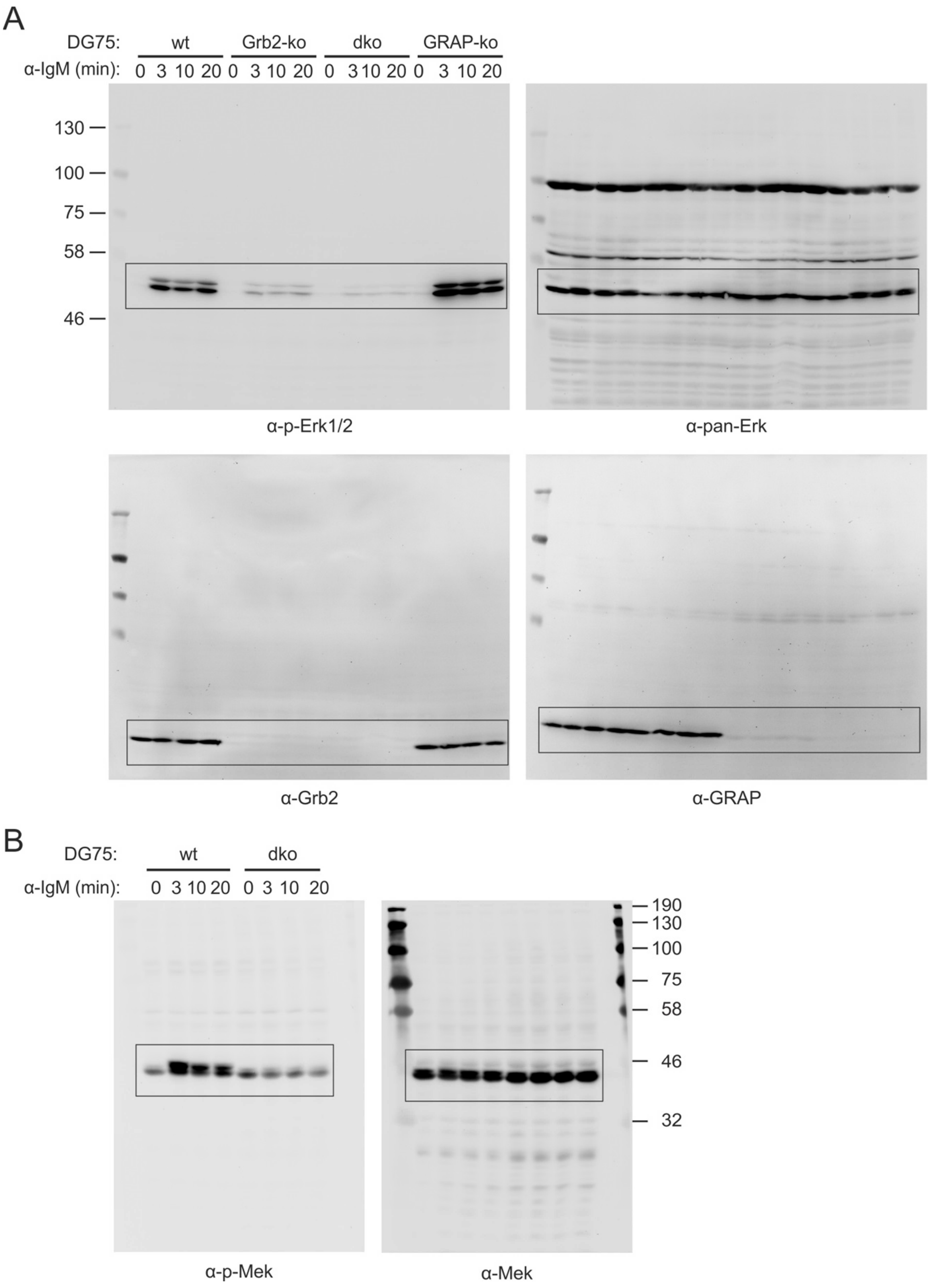

Supplementary Figure 11 | Uncropped western blots shown in Figure 3A (A) and in Figure 3B (B). Nitrocellulose membranes were sequentially developed from left to right (from top to bottom). The cropped parts are indicated by boxes.

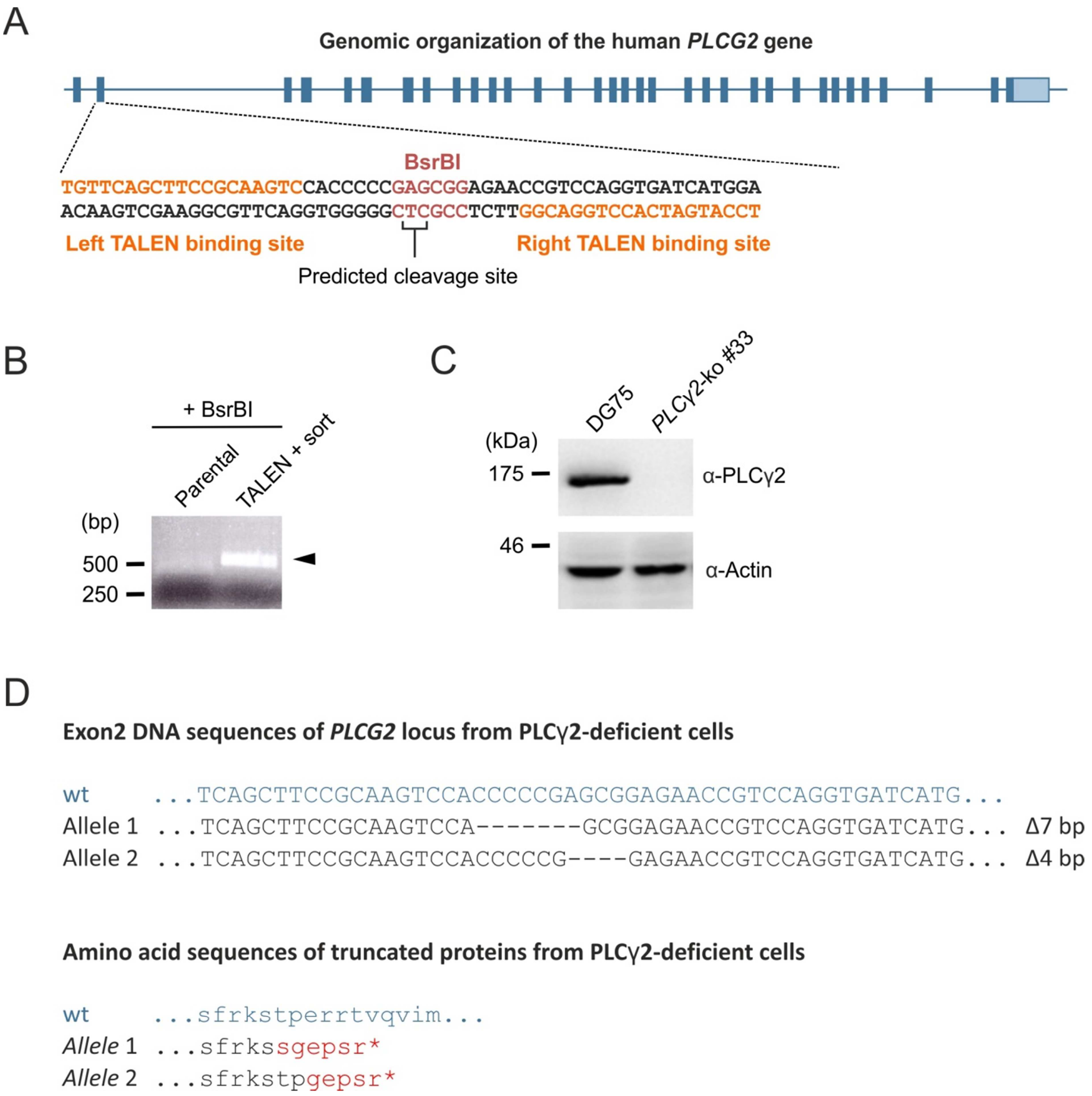

Supplementary Figure 12 | Generation of a PLCɣ2-deficient subline of DG75 B cells. (A) Schematic representation of the locus of the human *PLCG2* gene (not drawn to scale). The TALEN constructs were designed to introduce a double-strand break within exon 2. Any indel mutation resulting from erroneous DNA double strand break repair would destroy a BsrBI restriction site. (B) Genomic DNA from sorted cells that had expressed the TALEN constructs for several days (or untreated parental cells as control) was used to amplify exon 2 of the *PLCG2* gene. The amplicon was then cleaved with BsrBI to test for TALEN activity. The arrowhead indicates cleavage-resistant exon 2. (C) Immunoblot analysis of lysates from parental DG75 cells and PLCɣ2-deficient subclone #33. (D) DNA sequences showing nucleotide deletions of the mutant *PLCG2* alleles and the resulting amino acid sequences of truncated PLCɣ2 proteins.

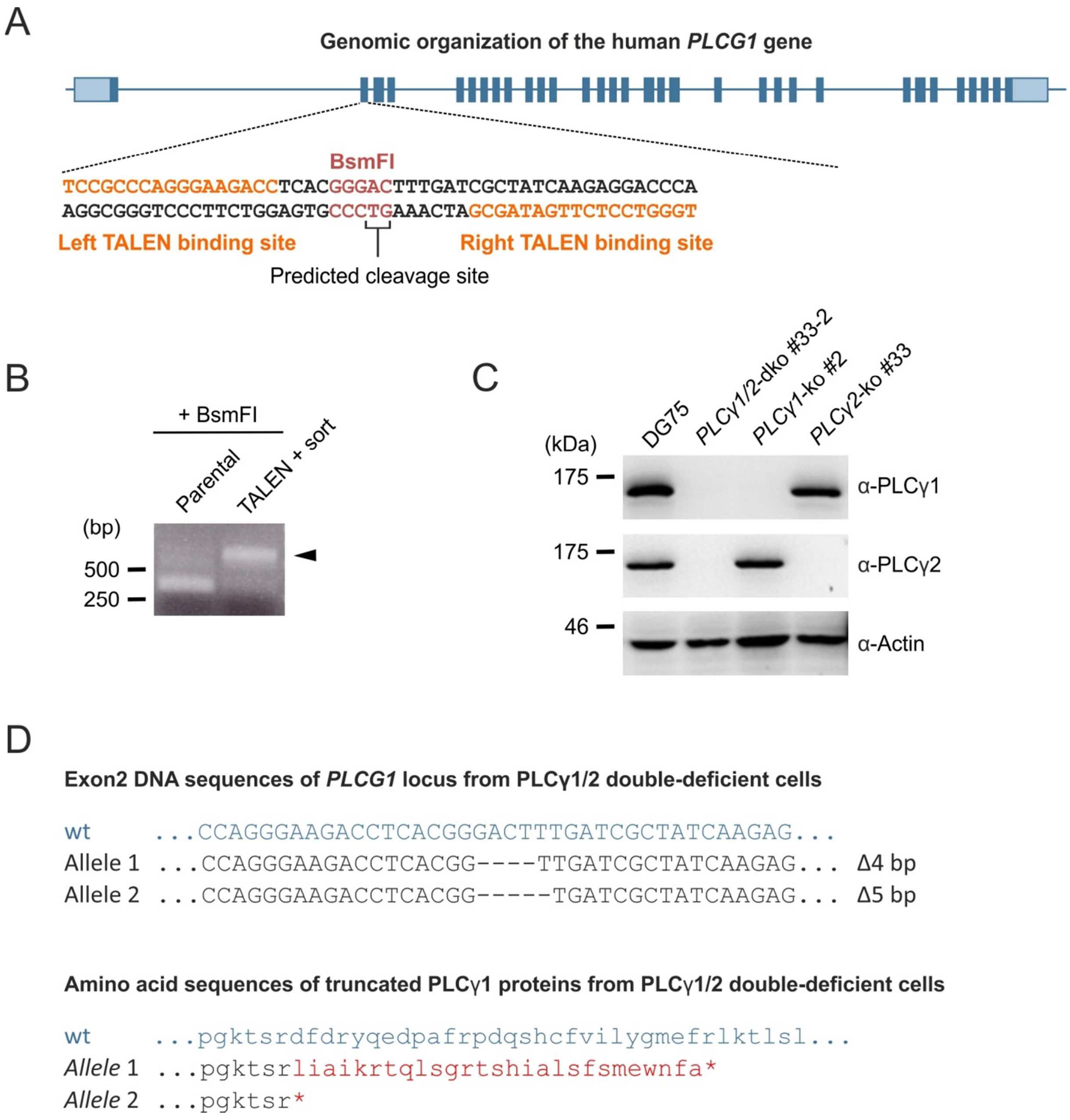

Supplementary Figure 13 | Generation of a PLCɣ2/PLCɣ1 double-deficient subline of DG75 B cells. (A) Schematic representation of the locus of the human *PLCG1* gene (not drawn to scale). The TALEN constructs were designed to introduce a double-strand break within exon 2. Any indel mutation resulting from erroneous DNA double strand break repair would destroy a BsmFI restriction site. (B) Genomic DNA from sorted cells that had expressed the TALEN constructs for several days (or untreated parental cells as control) was used to amplify exon 2 of the *PLCG1* gene. The amplicon was then cleaved with BsmFI to test for TALEN activity. The arrowhead indicates cleavage-resistant exon 2. (C) Immunoblot analysis of lysates from the indicated DG75 variants. (D) DNA sequences showing nucleotide deletions of the mutant *PLCG1* alleles and the resulting amino acid sequences of truncated PLCɣ1 proteins.

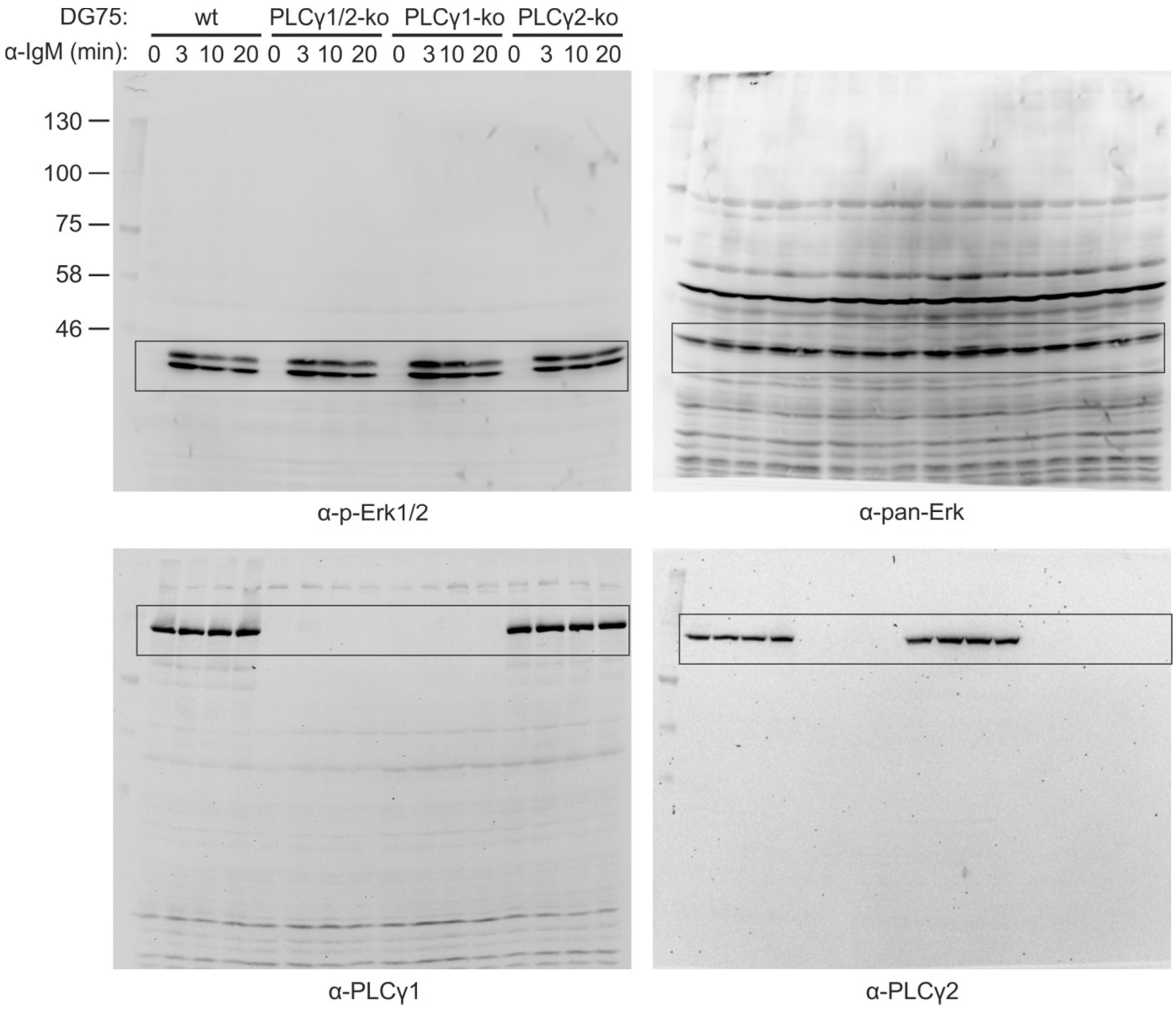

Supplementary Figure 14 | Uncropped western blots shown in Figure 4B. Nitrocellulose membrane was sequentially developed from left to right (from top to bottom). The cropped parts are indicated by boxes.

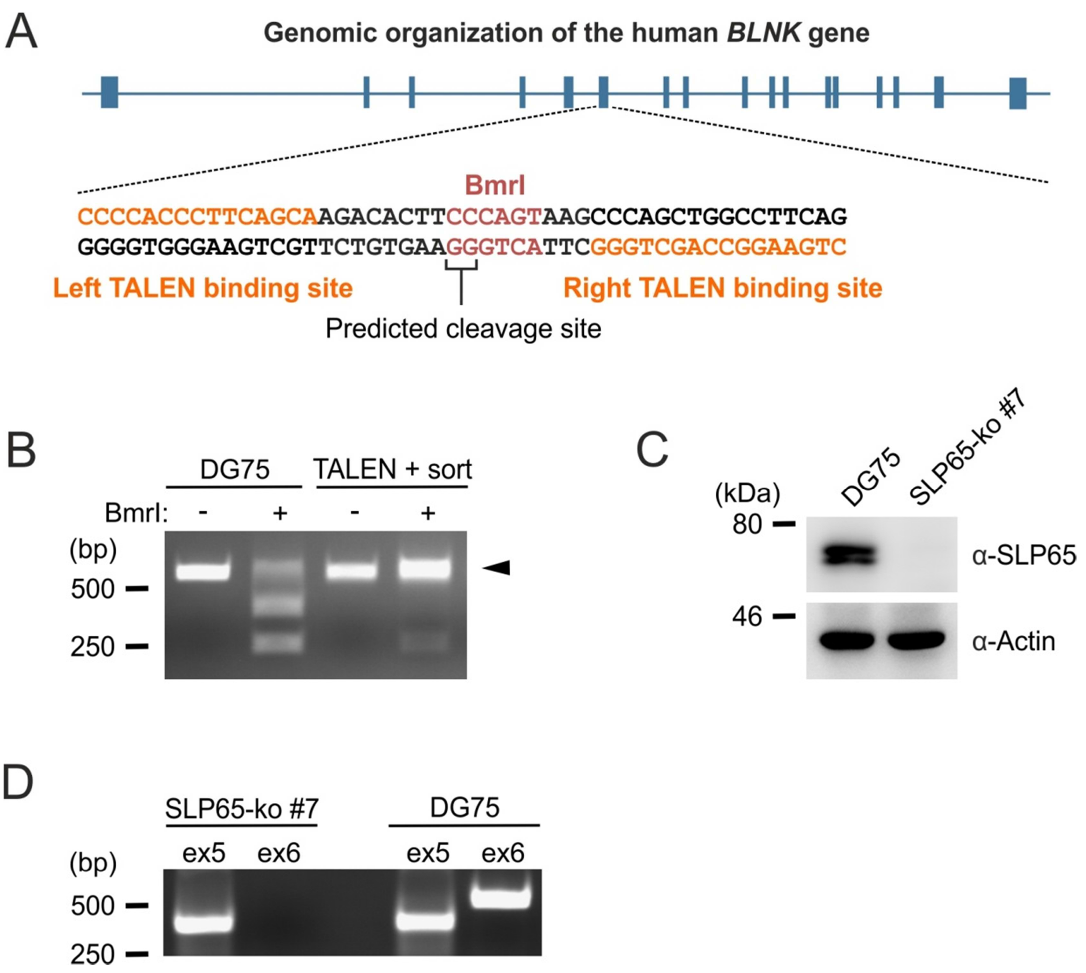

Supplementary Figure 15 | Generation of a SLP65-deficient subline of DG75 B cells. (A) Schematic representation of the locus of the human *BLNK* gene (encoding SLP65) (not drawn to scale). The TALEN constructs were designed to introduce a double-strand break within exon 6. Any indel mutation resulting from erroneous DNA double strand break repair would destroy a BmrI restriction site. (B) Genomic DNA from sorted cells that had expressed the TALEN constructs for several days (or untreated parental cells as control) was used to amplify exon 6 of the *BLNK* gene. The amplicon was then cleaved with BmrI to test for TALEN activity. The arrowhead indicates cleavage-resistant exon 6. (C) Immunoblot analysis of SLP65 expression in parental DG75 cells and SLP65-ko clone #7. (D) Genomic DNA of SLP65-ko clone #7 was used to amplify the TALEN-targeted exon 6 (ex6) of the *BLNK* locus. In contrast to parental DG75 cells, exon 6 could not be amplified from the SLP65-deficient cells, indicating the presence of large deletions on both alleles that prevent binding of at least one primer. As control, we amplified exon 5 (ex5) to verify the integrity of the used genomic DNA.

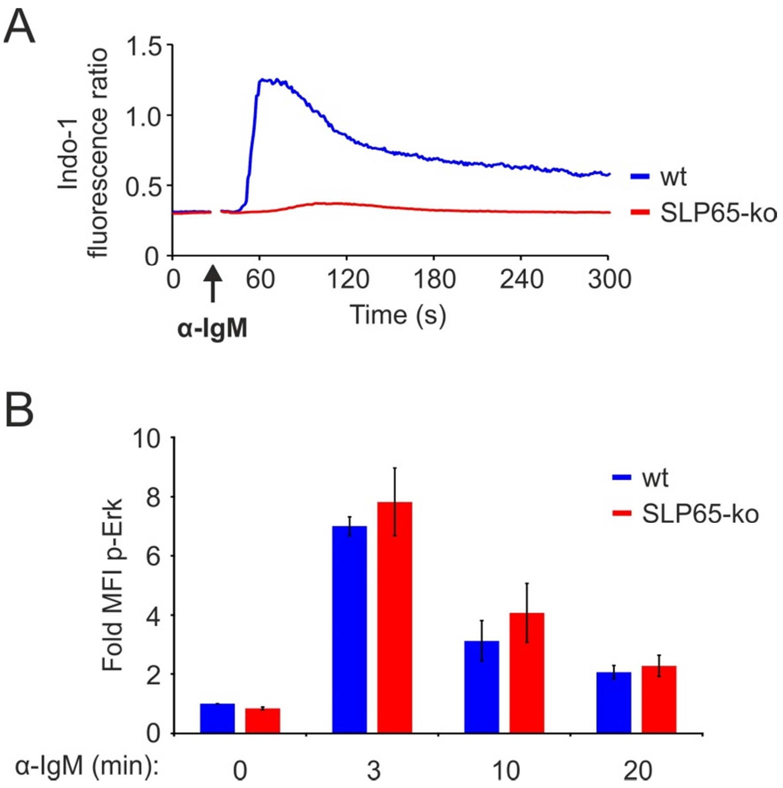

Supplementary Figure 16 | BCR-induced activation of Erk in human B cells does not require SLP65. (A) Analysis of BCR-induced Ca^{2+} mobilization in parental (blue curve) and SLP65-deficient (red curve) DG75 B cells on stimulation with anti-IgM $F(ab')_2$ fragments. (B) The kinetics of Erk activation in the same cells was determined by flow cytometric analysis of intracellularly stained phospho-Erk. Cells were either left untreated (0) or were stimulated via their mIgM-BCRs for the indicated time points. Basal signal intensities of unstimulated parental DG75 B cells (wt, blue bars) were defined as 1.0 and all other fluorescence intensities were normalized accordingly. Results for SLP65-deficient cells are shown as red bars. Error bars represent standard deviation of three independent experiments.

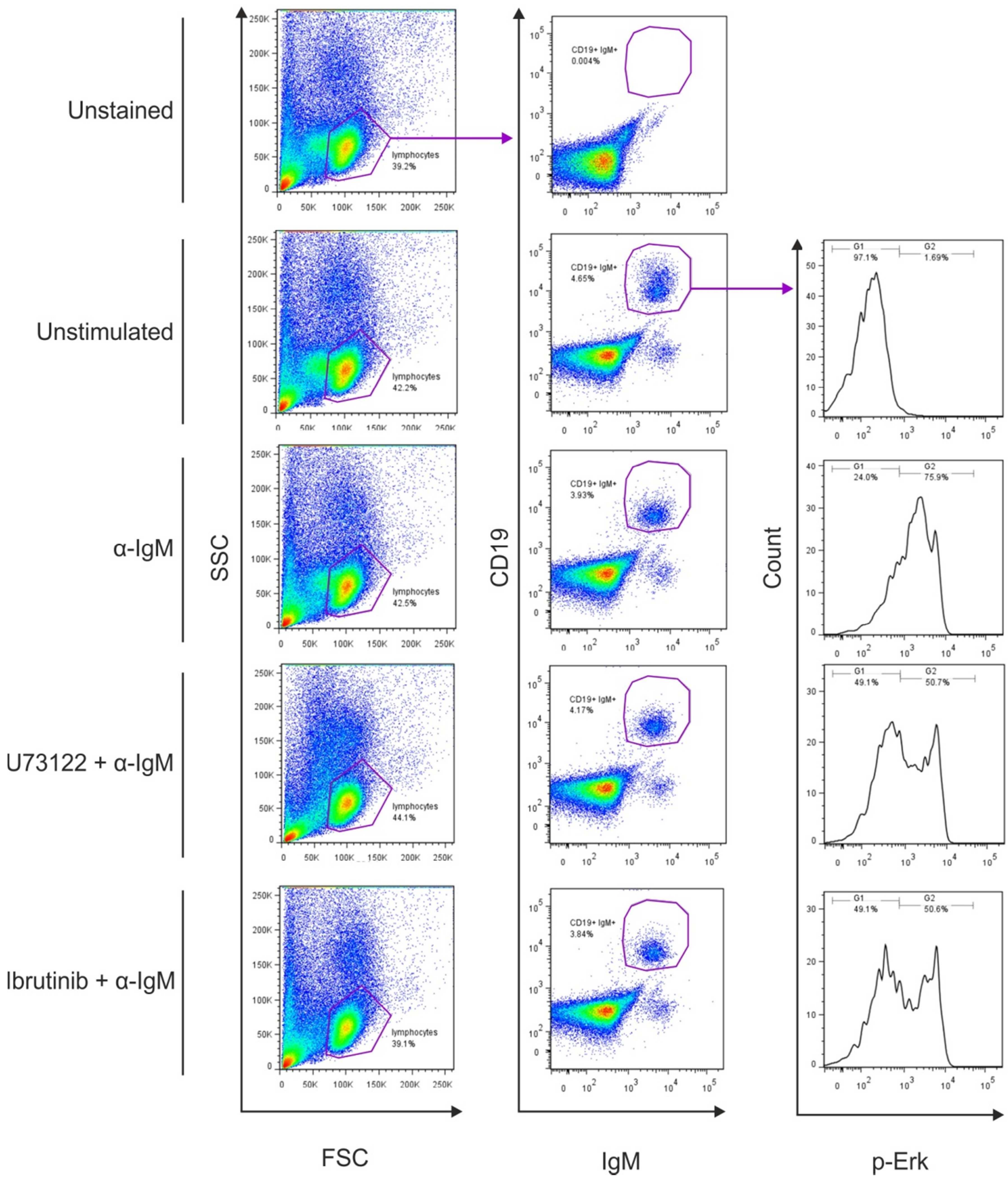

Supplementary Figure 17 | Appendix to Figure 6. Gating strategy to identify primary B cells for phospho-Erk analysis is shown for healthy donor 1 (HD1).

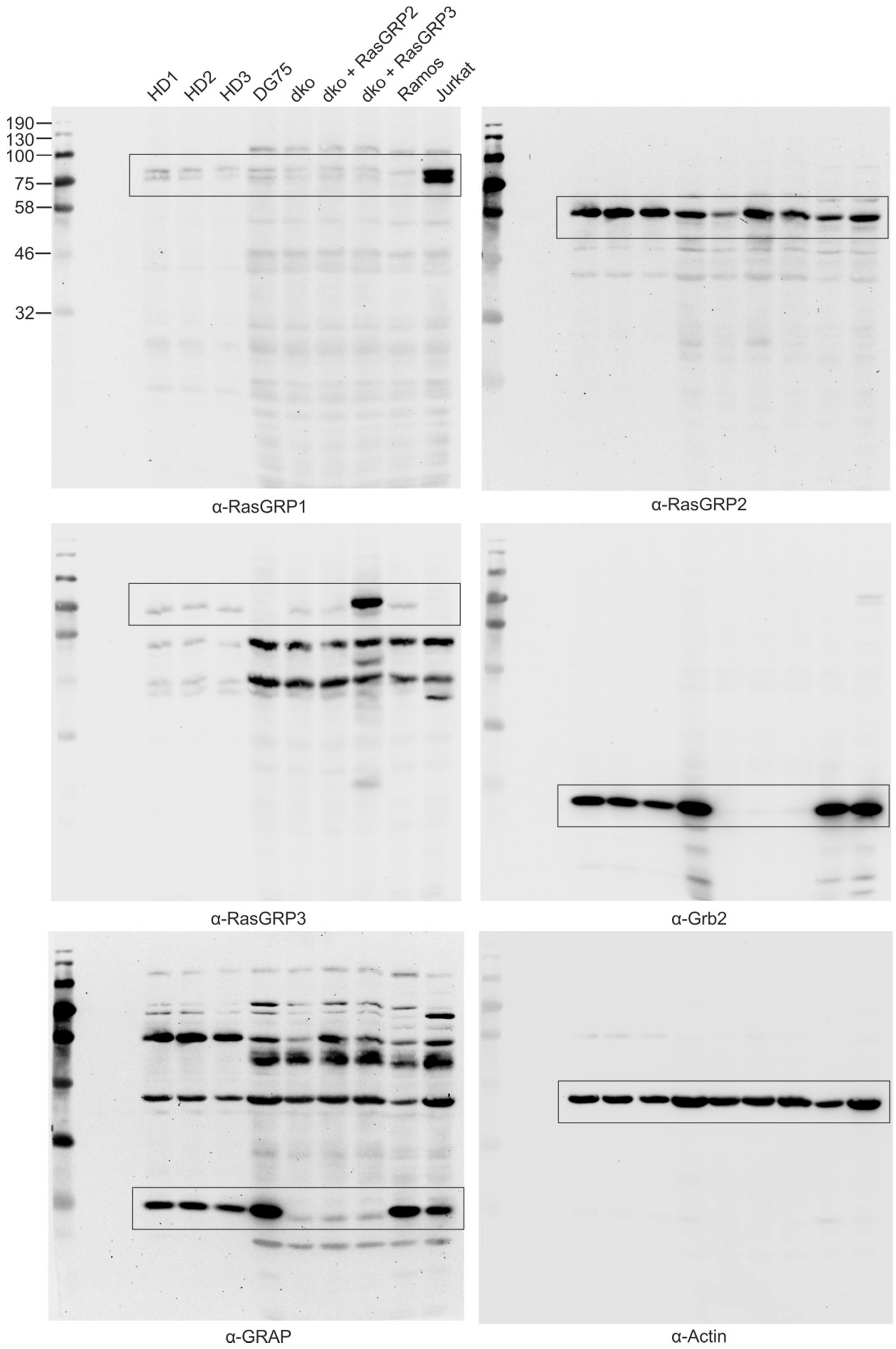

Supplementary Figure 18 | Uncropped western blots shown in Figure 6B. Nitrocellulose membrane was sequentially developed from left to right (from top to bottom). The cropped parts are indicated by boxes.

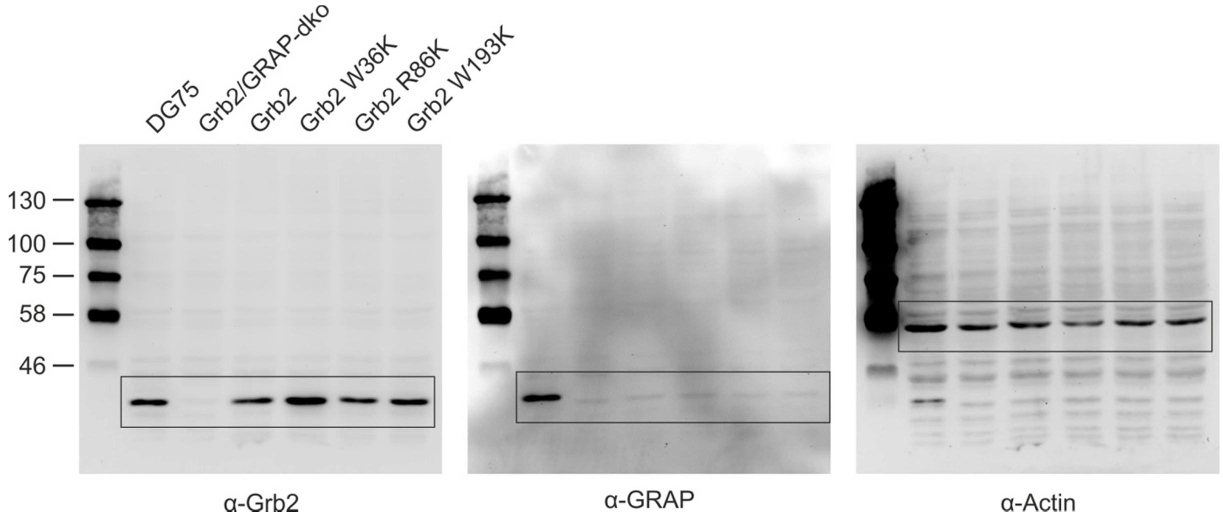

Supplementary Figure 19 | Uncropped western blots shown in Figure 7B. Nitrocellulose membrane was sequentially developed from left to right. The cropped parts are indicated by boxes.

A

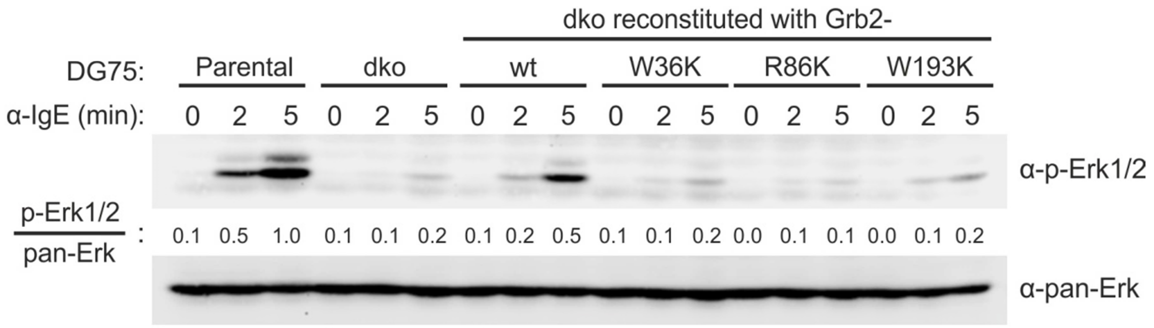

B

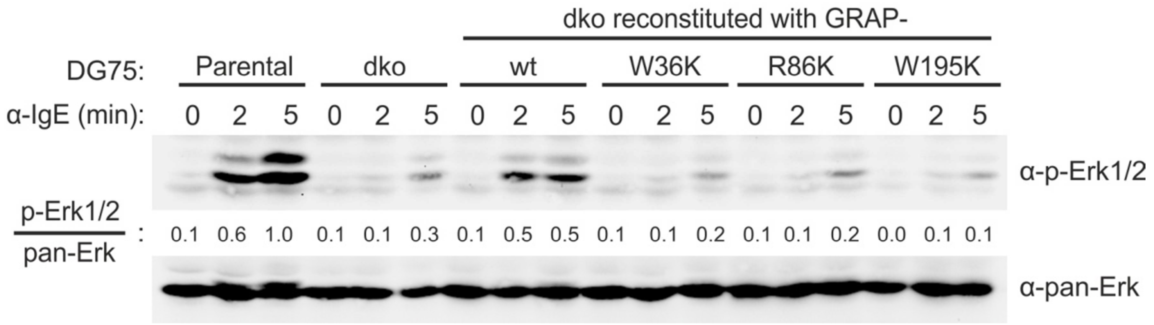

Supplementary Figure 20 | Erk activation on stimulation of the mIgE-BCR requires Grb2 or GRAP. Activation of Erk was analyzed in DG75 cells either lacking Grb2 and GRAP (Grb2/GRAP-dko) or expressing wild type (wt) or mutant variants of Grb2 (A) or GRAP (B) following stimulation with 10 µg/ml anti-IgE antibodies for the indicated times. Parental DG75 cells served as additional control. Activation of Erk kinases following stimulation of wild type mIgE-BCRs was analyzed by immunoblotting of cleared cellular lysates using antibodies to phospho-Erk (α-p-Erk1/2) and non-phosphorylated Erk (α-pan-Erk). Band intensities were quantified and the ratio of signal intensities for phospho-Erk divided by the signal intensities for total Erk is given. Maximal Erk activation was set to 1 and all other signal intensities were calculated accordingly.

A

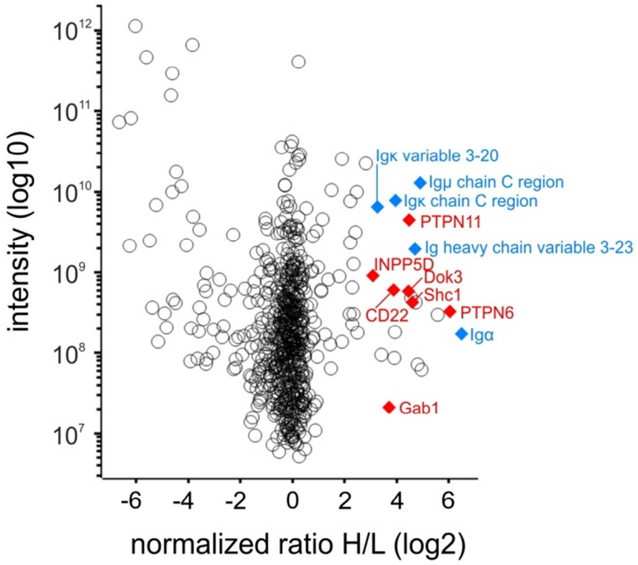

B

	Normalized ratio (H/L)	No. Peptides	Gene name	Protein ID	Protein name
BCR complex	89.6	2	CD79A	P11912	Igα
	30.12	5	IGHM	A0A075B6N9	Ig μ chain C region
	26.06	4	IGHV3-23	P01765	Ig heavy chain variable 3-23
	15.44	13	IGKC	A0A087X130	Ig κ chain C region
	9.63	9	IGKV3-20	P04206	Ig κ variable 3-20
Signaling	65.81	11	PTPN6	P29350	Tyrosine-protein phosphatase non-receptor type 6
	28.2	4	STAM2	O75886	Signal transducing adapter molecule 2
	24.52	12	SHC1	P29353-2	SHC-transforming protein 1
	22.18	4	PTPN11	Q06124	Tyrosine-protein phosphatase non-receptor type 11
	22.03	9	DOK3	D6RAM3	Docking protein 3
	15.14	6	HGS	O14964-2	Hepatocyte growth factor-regulated tyrosine kinase substrate
	14.74	16	CD22	P20273-4	CD22
	13.13	4	GAB1	Q13480	GRB2-associated-binding protein 1
	8.53	11	INPP5D	Q92835-2	Phosphatidylinositol 3,4,5-trisphosphate 5-phosphatase 1
Endocytosis & trafficking	26.7	10	EPS15L1	Q9UBC2-4	Epidermal growth factor receptor substrate 15-like 1
	23.65	14	ITSN2	Q9NZM3-2	Intersectin-2
	10.62	3	SNX18	Q96RF0-3	Sorting nexin-18
	5.56	8	CLTC	Q00610-2	Clathrin heavy chain 1
	5.35	12	DST	Q03001-3	Dystonin
DNA replication	7.05	4	RPA1	P27694	Replication protein A 70 kDa DNA-binding subunit
	5.61	15	TOP3A	Q13472-2	DNA topoisomerase 3-alpha
	5.08	3	BLM	P54132	Bloom syndrome protein
	5.05	2	HERC2	O95714	E3 ubiquitin-protein ligase HERC2
Ubiquitination	30.98	1	FAM175B	Q15018	BRISC complex subunit Abro1
	15.32	8	ANKRD13A	Q8IZ07	Ankyrin repeat domain-containing protein 13A
Unknown	47.46	5	CASC4	Q6P4E1-3	Protein CASC4

Supplementary Figure 21 | Identification of interaction partners of the Grb2 SH2 domain. (A) Dot plot of the identified proteins in the mass spectrometric analysis. All identified proteins are plotted according to their ‘heavy’ versus ‘light’ ratio of enrichment (H/L) on logarithmic scales. Proteins with H/L ratio > 4 were defined as specific interaction partners of the Grb2 SH2 domain. Proteins that were previously described to be involved in the BCR pathway are highlighted in red, components of the BCR are indicated in blue. (B) List of proteins that were identified as interaction partners of the Grb2 SH2 domain, grouped based on function. In each group proteins are sorted from top to bottom according to the normalized ratio of enrichment.

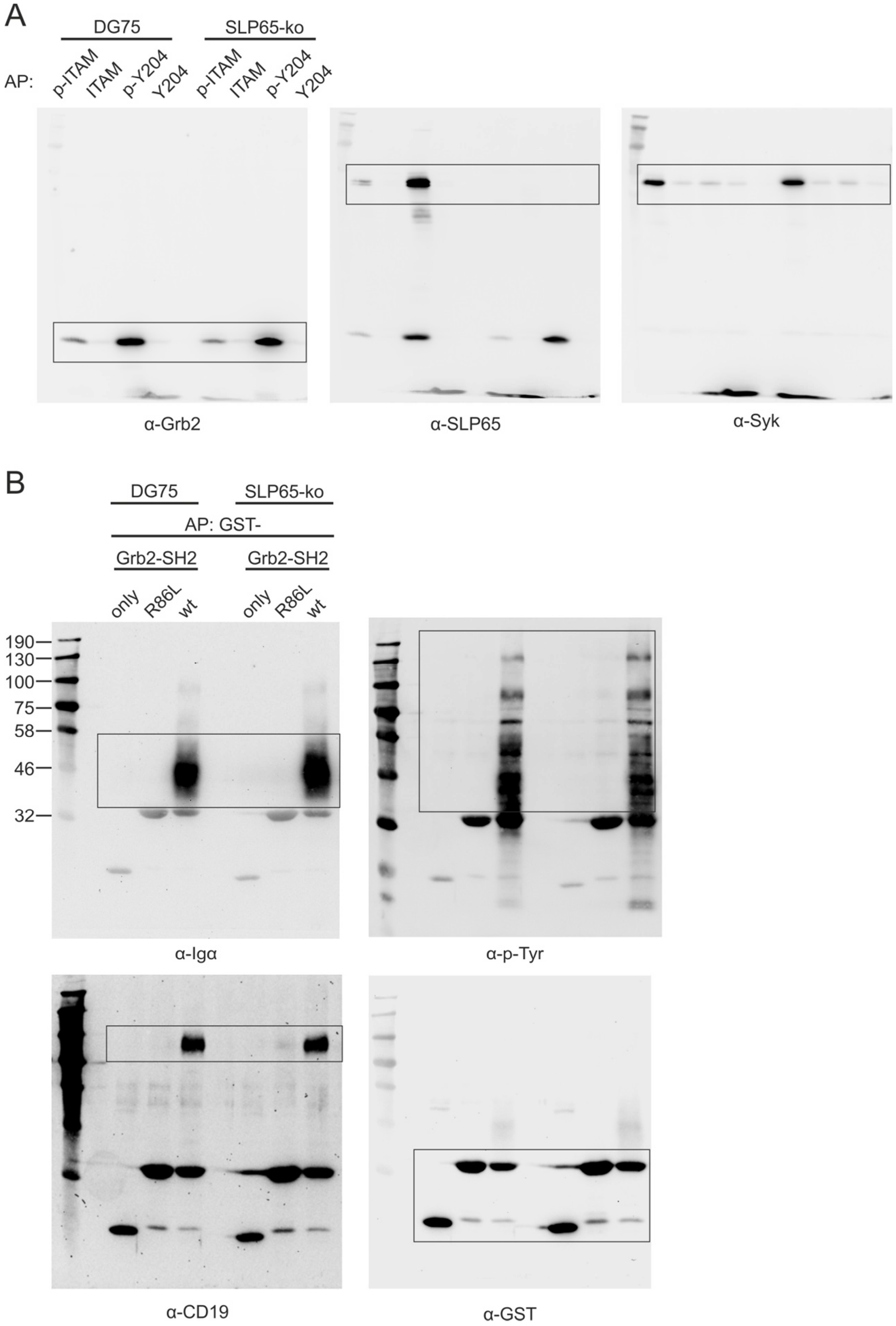

Supplementary Figure 22 | Uncropped western blots shown in Figure 8A (A) and 8B (B). Nitrocellulose membranes were sequentially developed from left to right (from top to bottom). The cropped parts are indicated by boxes.

Supplementary References

1 Engels, N. *et al.* The immunoglobulin tail tyrosine motif upgrades memory-type BCRs by incorporating a Grb2-Btk signalling module. *Nat Commun* **5**, 5456, doi:10.1038/ncomms6456 (2014).
